# YBX1 regulation of alveolar type II epithelial cells in idiopathic pulmonary fibrosis: mechanistic insights and small-molecule drug screening

**DOI:** 10.1186/s12967-025-07297-2

**Published:** 2025-11-18

**Authors:** Yijie He, Jin Li, Yibo Xie, Yaming Wu, Li Wang, Jungang Ren, Zhiqiang Zhang, Tong Yu, Shuxia Jiang, Hongli Shan, Yun Wu, Yuhong Zhou

**Affiliations:** 1 Department of Basic Medicine, Key Laboratory Of Functional and Clinical Translational Medicine, Xiamen Medical College, Xiamen, Fujian, 361023 P. R. China; 2 Department of Basic Medicine, Institute of Respiratory Research, Xiamen Medical College, Xiamen, Fujian, 361023 P. R. China; 3 Department of Neurology, The Second Affiliated Hospital of Harbin Medical University, Harbin, Heilongjiang 150081 P. R. China; 4 Shanghai Frontiers Science Research Center for Druggability of Cardiovascular Noncoding RNA, Institute for Frontier Medical Technology, Shanghai University of Engineering Science, Shanghai, 201620 P. R. China; 5 Department of Pharmacy, Xiamen Medical College, Xiamen, Fujian, 361023 P. R. China

**Keywords:** YBX1, Idiopathic pulmonary fibrosis, Alveolar type II epithelial cells, Immune microenvironment, Virtual screening, Molecular dynamics simulation

## Abstract

**Background:**

This study aims to systematically elucidate the molecular mechanisms underlying idiopathic pulmonary fibrosis (IPF), with a specific focus on the regulatory role of the nucleic acid-binding protein Y-box binding protein 1 (YBX1) in alveolar type II epithelial cells (AT2) and its association with disease progression. Additionally, the study integrates virtual screening and molecular dynamics (MD) simulations to identify small-molecule compounds targeting YBX1, thereby providing both mechanistic insights and therapeutic candidates for IPF.

**Methods:**

We employed integrative multi-omics analysis and bioinformatics approaches to identify IPF-associated signature genes, construct a diagnostic model and risk scoring system, and establish YBX1 as a central regulatory node. Single-cell RNA sequencing (scRNA-seq) data were used to characterize AT2 cell heterogeneity and developmental trajectories, highlighting the dynamic expression pattern of YBX1 during cell fate transitions. Cell–cell communication analysis elucidated YBX1’s potential involvement in immunomodulatory signaling, particularly between AT2 cells and M2 macrophages. Mendelian randomization was applied to infer the causal relationship between YBX1 expression and lung function indices. The expression and functional role of YBX1 were further validated using independent clinical cohorts and in vitro cell models. Structure-based virtual screening was performed to identify candidate compounds targeting YBX1, followed by MD simulations to assess binding stability and infer potential mechanisms of action.

**Results:**

YBX1 emerged as a key molecular signature of IPF with strong diagnostic potential and a prominent role in modulating immune cell infiltration. scRNA-seq revealed significant AT2 cell subtype diversity, with YBX1 dynamically expressed along differentiation trajectories. Intercellular communication analysis suggested that YBX1 may mediate indirect signaling between AT2 cells and M2 macrophages, potentially influencing the immune microenvironment and fibrotic progression. Mendelian randomization supported a significant positive causal relationship between YBX1 expression and pulmonary function, suggesting a protective role. Both clinical samples and cell-based assays confirmed YBX1 downregulation in fibrotic lung tissue, and its restoration improved mitochondrial function and enhanced antioxidant capacity. Virtual screening identified several small molecules with high binding affinity to functional domains of YBX1. MD simulations further supported the structural stability of YBX1–ligand complexes and suggested conformational regulation as a potential mechanism of action.

**Conclusion:**

This study delineates the pivotal role of YBX1 in the pathogenesis of IPF, highlighting its function in maintaining alveolar epithelial cell homeostasis and regulating disease progression. The identification of YBX1-targeting compounds through virtual screening and MD simulations offers a rational framework for the development of targeted therapies, advancing the translational potential of YBX1 as a diagnostic and therapeutic target in IPF.

**Supplementary Information:**

The online version contains supplementary material available at 10.1186/s12967-025-07297-2.

## Introduction

Idiopathic pulmonary fibrosis (IPF) is a progressive interstitial lung disease of unknown etiology, characterized by excessive fibrosis of the alveolar interstitium, leading to structural disruption of lung tissue and irreversible decline in pulmonary function [1–4]. The insidious onset and unclear cause of IPF result in progressive dyspnea and impaired lung capacity, severely compromising patients’ quality of life [5]. Prior to 2010, no approved therapy existed for this devastating disease. Even today, therapeutic options remain limited, and current antifibrotic agents, such as pirfenidone and nintedanib, fail to effectively halt disease progression [6]. Lung transplantation remains the only definitive intervention for advanced IPF [7]. These limitations underscore the urgent need to elucidate the pathogenic mechanisms of IPF and to identify novel diagnostic biomarkers and therapeutic targets to improve clinical outcomes and advance precision medicine.

The pathogenesis of IPF involves a complex interplay of diverse cell types and aberrant molecular signaling pathways. Among these, alveolar type II epithelial cells (AT2) are critical for maintaining alveolar integrity, not only through surfactant secretion but also by facilitating alveolar repair and regeneration [8]. Accumulating evidence indicates that AT2 cell injury, dysfunction, and aberrant repair processes are central drivers of IPF progression [9]. A deeper understanding of the molecular regulation governing AT2 cell behavior is thus essential for uncovering key pathological mechanisms and informing the development of targeted therapeutic strategies.

Abnormal regulation of gene expression is recognized as a crucial contributor to the molecular pathology of IPF. Alternative RNA splicing, a fundamental mechanism for generating transcriptomic and functional diversity, plays a pivotal role in various fibrotic and pulmonary diseases [10–12]. Splicing factors—core regulators of pre-mRNA splicing—modulate cellular signaling and metabolic pathways by directing the selective inclusion of exons. Recent studies have revealed substantial alterations in RNA splicing patterns in IPF lung tissues, closely linked to fibrotic progression, suggesting that splicing factors may play a pathogenic role in disease development [13, 14]. Y-box binding protein 1 (YBX1), a multifunctional nucleic acid-binding protein involved in transcriptional regulation, mRNA splicing, translational control, and cellular stress responses [15], has emerged as a key modulator in multiple pathological contexts, including tumorigenesis and fibrosis [16–18]. However, the precise functional role and regulatory mechanisms of YBX1 in IPF—particularly within AT2 cells—remain largely undefined. Given its involvement in cellular metabolism and survival, YBX1 may represent a critical node in IPF pathophysiology warranting comprehensive investigation.

The rapid advancement of multi-omics integration and single-cell RNA sequencing (scRNA-seq) technologies has revolutionized the study of complex diseases [19–21]. While bulk transcriptomic analyses provide insights into global gene expression changes, they lack the resolution needed to dissect cell-type-specific heterogeneity and function within diseased tissues. In contrast, scRNA-seq enables high-resolution identification of cellular subpopulations, developmental trajectories, and intercellular communication networks, offering a more nuanced understanding of IPFs pathological heterogeneity [22]. Coupled with machine learning algorithms applied to bulk transcriptomic data, this approach facilitates the robust identification of key disease genes and regulatory networks [23–31], providing a foundation for precision diagnostics and prognostic modeling.

In the IPF immune microenvironment, the infiltration and activation of M2-polarized macrophages and other immune cells actively contribute to fibrotic progression [32, 33]. Investigating intercellular signaling and communication pathways between immune cells and AT2 cells is crucial for elucidating YBX1’s potential immunomodulatory functions. Recent single-cell studies have identified unique AT2 subpopulations, such as AT2CCL20, that are closely associated with immune infiltration and correlate with poor prognosis in IPF patients, highlighting the functional importance of AT2–immune cell interactions [34]. Moreover, genetic epidemiological approaches like Mendelian randomization (MR) provide robust evidence for causal links between gene expression and clinical phenotypes. A recent genomewide MR study in IPF patients identified several druggable genes whose expression is causally related to lung function decline (FVC and DLCO), supporting the use of such methods in target validation [35].

In the context of drug discovery, structure-based virtual screening coupled with molecular dynamics (MD) simulations has become an effective strategy for identifying and optimizing novel therapeutic agents [36]. By constructing a three-dimensional structural model of the YBX1 protein and performing high-throughput virtual screening, we can identify small-molecule candidates with strong binding affinity. Subsequent MD simulations enable evaluation of the stability and interaction dynamics of YBX1–ligand complexes, thereby laying the groundwork for rational drug design targeting YBX1.

**Fig. 1:**
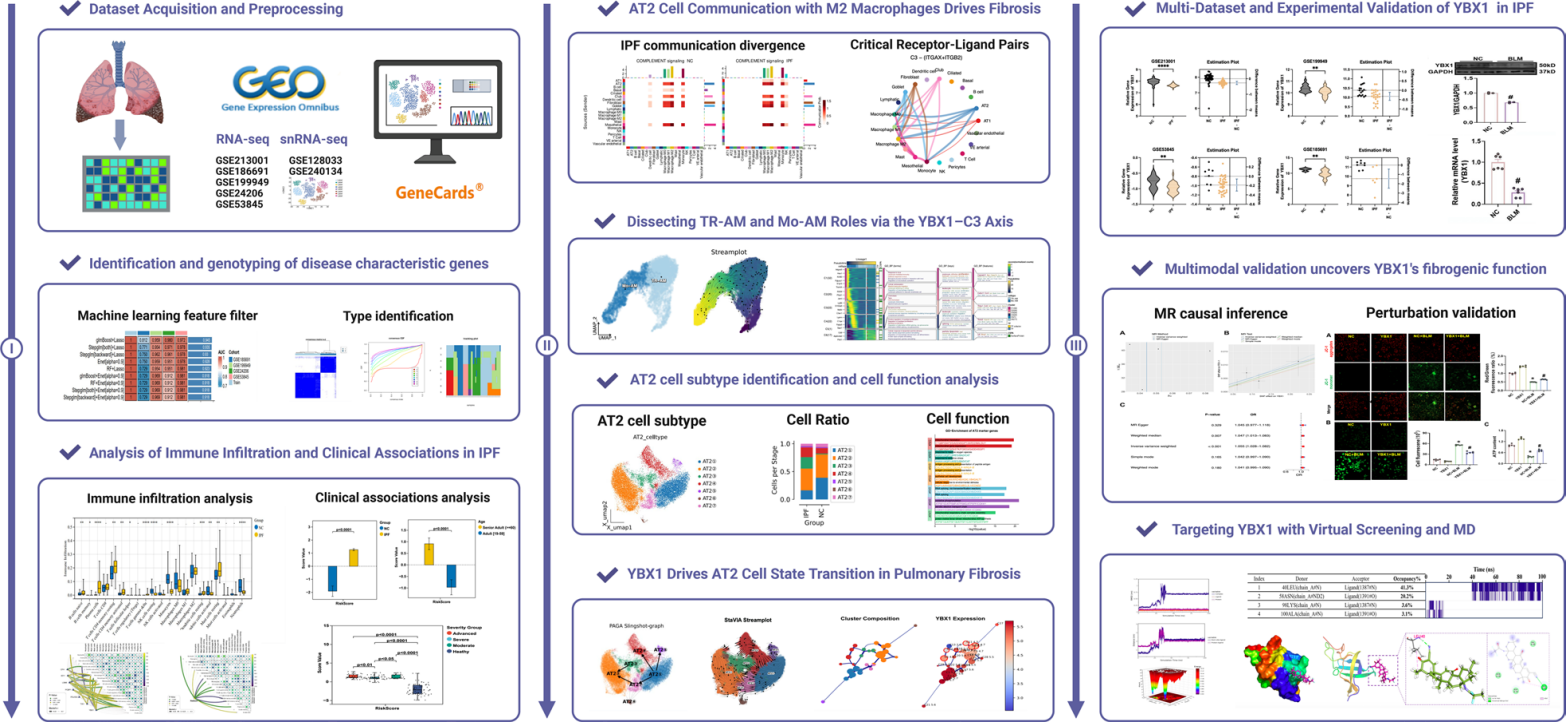
Integrated multi-omics analysis, computational drug screening, and experimental validation of YBX1 in pulmonary fibrosis

In summary, this study integrates multi-omics analyses and machine learning approaches to prioritize YBX1 as a candidate regulator in IPF. Leveraging single-cell transcriptomics, we dissect the heterogeneity and developmental trajectories of AT2 cells and delineate YBX1’s role in cell fate determination and immune regulation. Additionally, virtual screening and MD simulations are employed to identify potential small-molecule modulators of YBX1. Collectively, this integrative framework offers mechanistic insights into IPF pathogenesis and presents innovative strategies for developing targeted therapies, with the ultimate goal of advancing clinical diagnosis and treatment (Fig. 1).

## Methods

### Data acquisition

We retrieved array-based gene expression data, bulk RNA-seq data, and single-cell RNA-seq (scRNA-seq) data for human IPF lung tissue and alveolar type II epithelial (AT2) cells from the NCBI Gene Expression Omnibus (GEO; https://www.ncbi.nlm.nih.gov/geo/) (Supplementary Table 1). Array data were preprocessed with standard normalization and accompanied by probe annotation. All other datasets consisted of raw count data. Additionally, we queried the GeneCards database (https://www.genecards.org/) using the keyword “Splicing Factors” and filtered the results by a score threshold equal to or greater than the median, yielding a total of 637 splicing factor genes (Supplementary Table 2). We also integrated relevant clinical metadata, including age, sex, and disease status, from each dataset.

### Data processing and analysis

#### Analysis of array and RNA-seq data

##### Differential expression analysis

From the bulk RNA-seq dataset GSE213001, we extracted the expression profiles of the 637 splicing factors and constructed a custom expression matrix. Differential expression analysis was performed to compare IPF samples and normal controls (NC) using the *limma* package in R [37]. Genes with a fold change > 1.2 and adjusted *p*-value < 0.05 were considered differentially expressed genes (DEGs). Heatmaps were generated using the *pheatmap* package, and volcano plots were visualized using *ggplot2*.

##### Machine learning–based feature selection and diagnostic model construction

We applied 11 machine learning algorithms and evaluated 114 algorithmic combinations to identify diagnostic gene signatures. These algorithms included support vector machine (SVM), least absolute shrinkage and selection operator (Lasso), gradient boosting machine (GBM), random forest (RF), elastic net (Enet), partial least squares regression (*plsRglm*), ridge regression, generalized linear model boosting (glmBoost), linear discriminant analysis (LDA), XGBoost, and naive Bayes. The analysis proceeded as follows: (a) Expression profiles of differentially expressed splicing factors identified in GSE213001 were input into each of the 114 algorithm combinations, using 10-fold cross-validation to construct diagnostic models.(b) Model performance was validated in four independent external datasets (GSE186691, GSE199949, GSE24206, and GSE53845).(c) For each model, the area under the receiver operating characteristic curve (AUC) was calculated across all validation datasets. The combination yielding the highest mean AUC was selected for feature gene identification and final model construction.

##### Functional enrichment

To investigate the biological functions and pathway involvements of selected feature genes, we performed Gene Ontology (GO) enrichment analyses using the *clusterProfiler* package [38]. Feature genes were first converted to Entrez IDs prior to analysis. Enrichment was considered significant at adjusted *p* < 0.05.

##### Consensus clustering and riskscore construction based on feature genes

Consensus clustering was employed to identify potential molecular subgroups within the GSE213001 dataset using the *ConsensusClusterPlus* R package [39]. Based on expression patterns of the selected feature genes, this unsupervised approach defined putative biological subtypes referred to as “IPFclusters.” Resampling was used to assess the stability and robustness of clustering. Clusters were visualized via consensus matrix heatmaps, with higher consensus (dark blue) indicating stronger cluster cohesion. The optimal number of clusters (K) was determined based on the consensus heatmap, cumulative distribution function (CDF) curves, and delta area plots, which represent relative changes in CDF area between successive K values. To quantify patient-level molecular risk, a RiskScore was computed using principal component analysis (PCA) via the prcomp function. Principal components 1 and 2 (PC1 and PC2) were extracted to construct a composite feature score following a gene expression index (GGI)-like approach [40–42]:

$$RiskScore = \sum P C1i + PC2i$$

##### Immune cell infiltration analysis

Immune cell infiltration was estimated using CIBERSORT [43], an algorithm based on linear support vector regression that deconvolutes bulk transcriptomic data to infer the relative proportions of immune cell types. The LM22 signature matrix, which comprises predefined expression profiles of 22 leukocyte subsets, was applied as the reference. For each sample in the GSE213001 dataset, normalized gene expression values were input into CIBERSORT to calculate infiltration scores for all 22 immune cell types. The classification of macrophage subsets (M0, M1, and M2) was determined according to the LM22 transcriptomic signatures. Correlations between immune cell infiltration scores, the expression of selected feature genes, and the RiskScore were evaluated using Pearson correlation coefficients, and the results were visualized with correlation heatmaps.

##### Clinical feature correlation analysis

Samples were stratified based on available clinical characteristics, including age (≥60 vs. < 60 years) and disease severity (Healthy, Moderate, Severe, Advanced). To evaluate associations between RiskScore and clinical subtypes, we employed the Kruskal–Wallis test or Wilcoxon rank-sum test, as appropriate, to identify statistically significant differences in RiskScore distributions among groups.

#### scRNA-seq data analysis

##### Quality control and preprocessing

Prior to analysis, we performed rigorous quality control on the single-cell RNA-sequencing (scRNA-seq) data to remove potential doublets, low-quality cells, and lowly expressed genes. Filtering criteria included mitochondrial gene content, transcript count, the number of genes expressed per cell, and cell complexity metrics. Uniform preprocessing was applied across all samples and batches using the *omicverse* [44] and *scanpy* [45] pipelines. Cells were retained if they met the following thresholds: mitochondrial gene percentage < 30%, unique molecular identifier (UMI) count ≥500, and detected genes ≥250. Potential doublets were further identified and removed using the *sccomposite* algorithm [46] to minimize their confounding effects on downstream analyses. Batch effects were corrected using the *Harmony* algorithm [47], ensuring consistency across different experimental batches.

##### Dimensionality reduction, clustering, and cell type annotation

We conducted dimensionality reduction and clustering on the batch-corrected scRNA-seq dataset. Using the Harmony-adjusted principal component space (X_pca_harmony), a k-nearest neighbor (KNN) graph was constructed with 15 neighbors and 50 principal components. Uniform Manifold Approximation and Projection (UMAP) was then applied to visualize the cellular landscape in a reduced dimension. Cells were clustered using the Leiden algorithm. Cell type annotation was performed manually based on canonical marker genes, quality control metrics, and curated gene sets from CellMarker 2.0. Subtypes of AT2 cells were annotated using the same approach.

##### AUCell scoring

We employed the *AUCell* algorithm [48] to quantify the enrichment of input gene sets at the single-cell level. AUCell calculates the area under the recovery curve (AUC) to determine whether a critical subset of genes within a set is enriched in the expressed genes of individual cells. As this method is ranking-based, it is independent of expression units and normalization procedures. We applied AUCell to evaluate the distribution of activity scores for feature genes across AT2 cell subpopulations, thereby enhancing our understanding of their functional roles.

##### Pseudotime analysis

To explore the developmental trajectories of AT2 cells, we first used *CytoTRACE2* [49] to infer cellular differentiation potential. Analysis parameters included a batch size of 10,000, a smoothing factor of 1,000, and parallel computation enabled to enhance efficiency. The number of principal components was set to 50, with a fixed random seed of 14 to ensure reproducibility.

Subsequently, we applied the *TrajInfer* framework using both UMAP embeddings and Harmony-corrected principal components (X_pca_harmony) as input, retaining 50 components (n_comps = 50) for trajectory inference. The starting cell population was defined based on CytoTRACE2 results. Three trajectory inference algorithms— *Slingshot* [50], *Palantir* [51], and *StaVIA* [52]—were employed to reconstruct lineage progressions.

For *Slingshot*, we used three iterations to optimize developmental trajectory estimation, generating trajectory plots across epochs (num_epochs = 3) to ensure visual clarity and convergence. To further validate these trajectories, we performed topological inference using *PAGA* (partition-based graph abstraction) [53], which delineates intercellular transitions based on Slingshot-derived pseudotime.

In *Palantir*, 500 waypoints (num_waypoints = 500) were used to refine trajectory resolution. Palantir’s branching algorithm identified distinct developmental lineages, and we visualized cell trajectories annotated with pseudotime entropy and lineage affiliation.

To complement these analyses, we employed the *VIA* (Vector-based Inference of Assembly) algorithm, a graph-based method that integrates lazy-teleporting random walks and Markov Chain Monte Carlo (MCMC) optimization to robustly capture complex topologies, including multifurcating, disconnected, and cyclic trajectories. VIAs flexibility and robustness against preprocessing variability make it well-suited for high-dimensional single-cell data. Pseudotime and trajectory estimates from VIA were visualized in UMAP space and interpreted in conjunction with cell type annotations.

Lastly, to examine the dynamic behavior of genes of interest along inferred lineages, we conducted gene trend analysis. Gene expression values were imputed using *MAGIC*, and temporal expression dynamics were inferred based on pseudotime estimates from the various algorithms. Expression trajectories were visualized to highlight temporal gene regulation patterns.

##### Cell–cell communication analysis

To investigate potential intercellular communication events, we employed the *CellChat* package [54] in R. Separate *CellChat* objects were created for the NC and IPF cohorts to enable comparative analysis. Intercellular communication probabilities were inferred using the computeCommunProb function, while pathway-specific interactions were identified using computeCommunProbPathway. The *CellChatDB.human* reference database, encompassing all categories of ligand–receptor interactions, was used to guide predictions. Interactions involving fewer than 10 cells were excluded to enhance statistical robustness. The resulting interaction networks provided a comprehensive overview of the signaling landscapes across cell types and disease conditions.

##### scRNA-seq analysis of alveolar macrophages in the BLM

We analyzed a publicly available scRNA-seq dataset from a bleomycin-induced mouse pulmonary fibrosis model (GSE240134), which included wild-type (WT), day 14 (BLM14), and day 28 (BLM28) groups.

Quality control and preprocessing were performed according to the same thresholds described for human scRNA-seq data (section “Cell–cell communication analysis”), including mitochondrial gene percentage < 30%, UMI count ≥500, and detected genes ≥250. Potential doublets were identified and removed using the sccomposite algorithm, and batch effects were corrected with the Harmony algorithm.

Dimensionality reduction and clustering were carried out as described in section “scRNA-seq analysis of alveolar macrophages in the BLM”. Specifically, a k-nearest neighbor (KNN) graph was constructed using the Harmony-adjusted principal component space (15 neighbors, 50 principal components). Uniform Manifold Approximation and Projection (UMAP) was applied for visualization, and cell clustering was performed with the Leiden algorithm. Cell type annotation was performed manually based on canonical marker genes and curated references.

Trajectory inference was conducted using Slingshot and StaVIA. Differential gene expression analyses between clusters were performed with the Wilcoxon rank-sum test (adjusted *p* < 0.05). Gene Ontology enrichment analysis was implemented using clusterProfiler.

### Mendelian randomization analysis

In this study, we employed a two-sample Mendelian randomization (MR) framework [55] to evaluate the potential causal effect of *YBX1* gene expression (exposure; eQTL identifier: eqtl-a-ENSG00000065978) on pulmonary function indices, including forced expiratory volume in 1 second (FEV₁, ukb-a-337) and forced vital capacity (FVC, ukb-a-336). Single nucleotide polymorphisms (SNPs) significantly associated with *YBX1* expression (*p* < 5 × 10^− 8^) were selected as instrumental variables (IVs) from the corresponding eQTL GWAS. We removed SNPs exhibiting high linkage disequilibrium (r^2^ < 0.001, distance > 10 kb) and excluded those associated with potential confounders.

Next, we extracted the effect sizes of these IVs from the relevant GWAS datasets for FEV₁ and FVC. When a SNP was absent from the outcome dataset, a proxy SNP in high linkage disequilibrium (r^2^ > 0.8) was used. During data harmonization, we aligned the effect alleles to ensure directional consistency between exposure and outcome associations.

Causal inference was primarily conducted using the inverse-variance weighted (IVW) method, complemented by the weighted median estimator and MR-Egger regression for sensitivity analyses. We assessed heterogeneity using Cochran’s Q statistic and tested for directional pleiotropy via the MR-Egger intercept. A leave-one-out analysis was performed to evaluate the influence of individual SNPs. All analyses were conducted in R (version 4.2) using the *TwoSampleMR* package (v0.5.6), adhering strictly to the three core assumptions of MR [1]: the relevance assumption—that IVs are strongly associated with the exposure [2]; the independence assumption—that IVs are not associated with confounders; and [3] the exclusion restriction—that IVs influence the outcome solely through the exposure.

### Experimental validation

#### MLE-12 cell culture

The mouse alveolar type II epithelial cell line (MLE-12) was cultured in complete Dulbecco’s Modified Eagle Medium (DMEM) supplemented with 10% fetal bovine serum (FBS) and 1% penicillin-streptomycin. Cells were maintained in a humidified incubator at 37 °C with 5% CO₂.

#### Cell transfection

When MLE-12 cells reached 70–80% confluency, transfection was carried out following the experimental protocol. Plasmid DNA encoding *YBX1* was transfected at a final concentration of 1 ng/μL, while siRNA (si-YBX1 and negative control si-NC) was used at 5 nM/mL. Culture medium was first replaced with serum-free DMEM. Solutions A and B were prepared and cooled on ice for 5 minutes in light-protected 1.5 mL Eppendorf tubes. The solutions were then mixed, incubated at room temperature for 15 minutes, and added to the cell culture wells. After 6 hours of incubation, the transfection medium was replaced with fresh complete medium.

#### Quantitative real-time PCR (qRT-PCR)

Total RNA expression levels were quantified using SYBR Green I (04913914001; Roche, Basel, Switzerland). GAPDH was used as the internal reference gene. Primer sequences were as follows:

- *YBX1* forward: 5′-AAGGTCATCGCAACGAAGGTT-3′
- *YBX1* reverse: 5′-CAAATACGTCTTCCTTGGTGCA-3′
- *GAPDH* forward: 5′-AAGAAGGTGGTGAAGCAGGC-3′
- *GAPDH* reverse: 5′-TCCACCACCCAGTTGCTGTA-3′

#### Western blot analysis

Protein lysates were prepared using RIPA buffer (P0013B, Beyotime, Shanghai, China) and quantified via the BCA assay (P0009, Beyotime). Proteins were separated using SDS-PAGE and transferred onto PVDF membranes. Membranes were blocked with non-fat milk (BS102, Biosharp, China) and incubated overnight at 4 °C with primary antibodies. After washing, membranes were incubated with secondary antibodies at room temperature. The following primary antibodies were used: rabbit anti-YBX1 (Proteintech, Wuhan, China; 20,339–1-AP; 1:500) and mouse anti-GAPDH (Zhongshan Jinqiao, Beijing, China; TA-09; 1:1000). Detection and quantification were performed using Odyssey v1.2 software (LI-COR Biosciences, Lincoln, NE, USA).

#### Mitochondrial membrane potential (MMP) assessment

MMP was evaluated using JC-1 dye (C2006, Beyotime, Shanghai, China). Cells were incubated with JC-1 staining solution according to the manufacturer’s protocol. In healthy cells with high MMP, JC-1 forms aggregates emitting red fluorescence (610 ± 10 nm), whereas in cells with low MMP, JC-1 remains in monomeric form emitting green fluorescence (525 ± 10 nm). The red-to-green fluorescence intensity ratio was used to assess MMP integrity.

#### ATP production assay

Intracellular ATP levels were measured using an ATP detection kit (S0026, Beyotime). ATP standards were prepared in ice-cold distilled water at five different concentrations. For each sample, 50 μL of ATP detection working solution was added and incubated for 5 minutes, followed by the addition of 10 μmol/L ATP standard solution. After rapid mixing, relative luminescence units (RLU) were recorded using a luminometer, and ATP concentrations were calculated based on the standard curve.

#### Reactive oxygen species (ROS) measurement

Intracellular ROS levels were assessed using a ROS detection kit (S0033S, Beyotime). DCF fluorescence was used for quantification, based on the oxidation of 2′,7′-dichlorodihydrofluorescein diacetate (DCFH-DA) by intracellular ROS to fluorescent DCF. Cells were incubated in 24-well plates with DCFH-DA for 25 minutes at 37 °C. ROS levels were then visualized under a fluorescence microscope (Zeiss, Jena, Germany) at an excitation wavelength of 488 nm.

### High-throughput virtual drug screening based on structure-based docking and molecular dynamics simulations

#### Computer-aided virtual drug screening

A structure-based virtual screening approach was employed to identify potential FDA-approved small-molecule drugs that may interact with the Y-box binding protein 1 (YBX1). A library comprising 2,648 FDA-approved compounds, obtained from the DrugBank database, was subjected to molecular docking using AutoDock Vina (version 1.2.0) [56]. Prior to docking, the binding pockets of YBX1 were accurately identified using CB-DOCK2 [57], which provided the spatial coordinates of the most probable active sites to be used as the docking grid centers.

Each compound was docked automatically into the predicted active site using Vina’s scoring function, which estimates binding affinity based on predicted free energy of binding. Docking results were ranked according to binding energy, and top-performing compounds with the most favorable binding scores were selected as candidate molecules for subsequent molecular dynamics (MD) simulations to further assess interaction stability and binding mechanisms.

#### Molecular dynamics simulation

To further evaluate the binding stability and interaction mechanisms between selected small-molecule candidates and YBX1, molecular dynamics simulations were conducted using GROMACS 2022 [58]. The protein component was parameterized with the AMBER14SB force field, while small-molecule ligands were modeled using the General AMBER Force Field (GAFF). Solvation was performed using the TIP3P water model. Protein–ligand complexes were constructed by merging coordinate files and solvated within a periodic boundary box.

Bond lengths involving hydrogen atoms were constrained using the LINCS algorithm, and the integration time step was set to 2 fs. Long-range electrostatic interactions were calculated using the Particle-Mesh Ewald (PME) method with a cutoff distance of 1.2 nm. Non-bonded interactions were truncated at 10 Å, with neighbor lists updated every 10 steps. Temperature was maintained at 298 K using the velocity-rescaling thermostat (V-rescale), and pressure was regulated at 1 bar using the Berendsen barostat.

Prior to production runs, the system underwent 100 ps of NVT (constant volume and temperature) and NPT (constant pressure and temperature) equilibration to stabilize the complex. Production simulations were then conducted for 100 ns, with trajectory snapshots saved every 10 ps for downstream analysis.

Trajectory data were visualized and analyzed using VMD and PyMOL to evaluate structural stability metrics, including root-mean-square deviation (RMSD) and hydrogen bonding profiles. Additionally, binding free energies between protein and ligand were estimated using the Molecular Mechanics/Poisson–Boltzmann Surface Area (MM/PBSA) method via the *g_mmpbsa* tool, providing thermodynamic validation of binding affinity and complex stability.

## Results

### Machine learning model development and feature gene selection

**Fig. 2:**
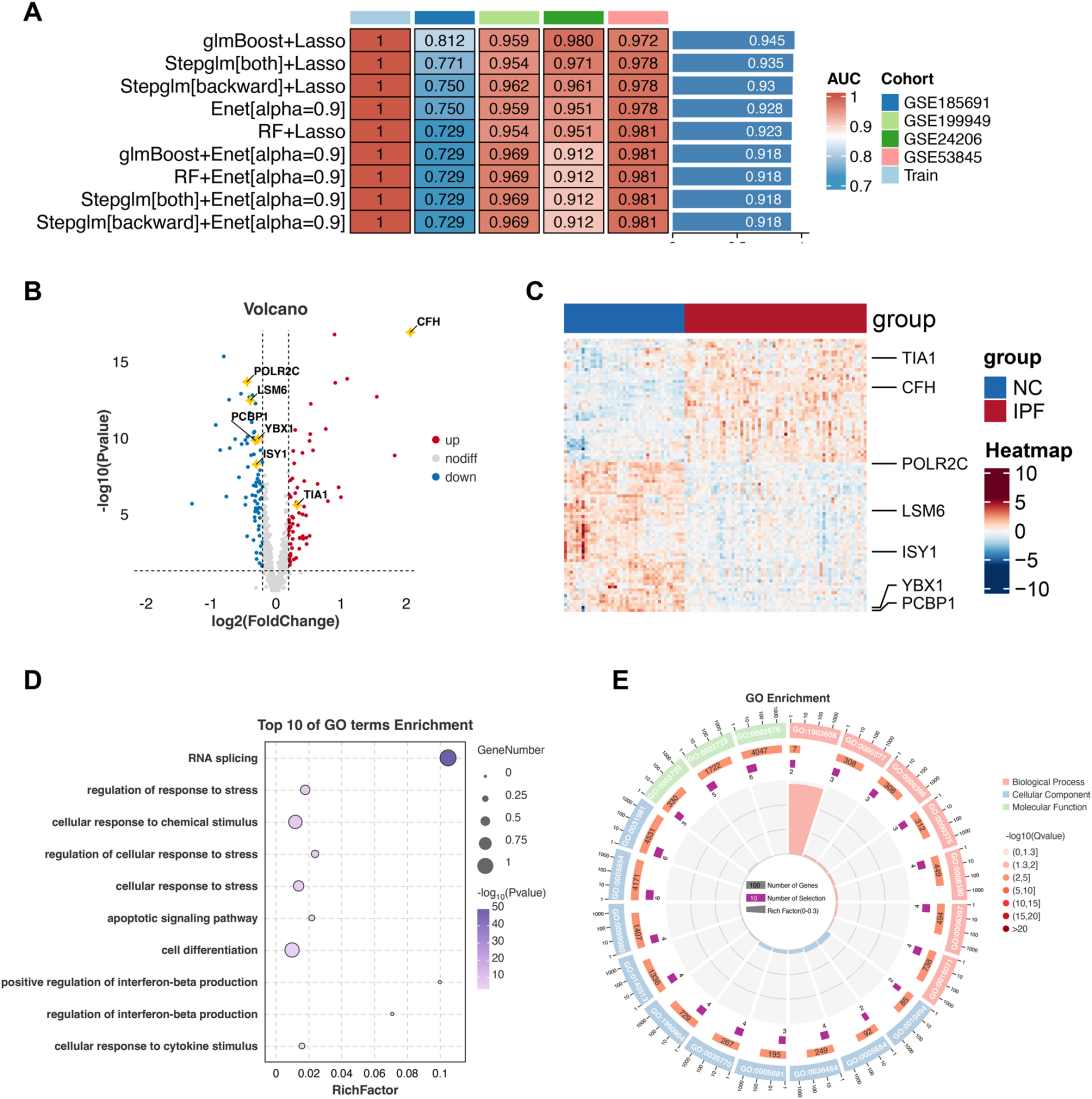
**A**: Performance of various machine learning models and their combinations evaluated using AUC across multiple cohorts (GSE185691, GSE199949, GSE24206, GSE53845, and the training set). **B**: Volcano plot showing the distribution of differentially expressed genes between IPF and normal control groups. Red points represent significantly upregulated genes, blue points represent significantly downregulated genes, and yellow stars highlight key feature genes selected for further analysis. **C**: Heatmap illustrating the expression profiles of the seven feature genes across normal controls (NC) and IPF patients. **D**: Bubble plot showing enriched GO terms. Dot size represents gene count and color intensity indicates -log10(p value). **E**: Chord diagram displaying gene-pathway associations. Outer ring colors denote GO categories (pink: biological process; blue: cellular component; green: molecular function), while inner purple segments show the proportion of target genes in each pathway

To identify key feature genes and construct a diagnostic model for IPF, we employed an integrated machine learning strategy. Multiple algorithmic combinations were evaluated (Fig. 2A), and their diagnostic performance was assessed across several datasets, including GSE185691, GSE199949, GSE24206, GSE53845, and the training cohort (GSE213001). Among all combinations, the glmBoost + Lasso model achieved the highest area under the curve (AUC) values across all datasets, with an average AUC of 0.945 in the training set and robust performance in independent validation cohorts.

Based on this model, we identified seven optimal feature genes—Complement Factor H(**CFH**), ISY1 Splicing Factor Homolog (**ISY1**), LSM6 Homolog (**LSM6**), Poly RC Binding Protein 1(**PCBP1**), RNA Polymerase II Subunit C(**POLR2C**), TIA1 Cytotoxic Granule Associated RNA Binding Protein (**TIA1**), and **YBX1**—for further investigation. A volcano plot (Fig. 2B) and heatmap (Fig. 2C) illustrate the differential expression of these genes between IPF and normal control (NC) groups.

To elucidate the biological significance of the selected feature genes, we performed Gene Ontology (GO) enrichment analysis directly on this gene set. The enrichment analysis (Fig. 2D–E) revealed associations with biological processes relevant to IPF pathogenesis, including **RNA splicing, stress responses, apoptotic signaling, and cytokine-mediated pathways**. While these genes are not themselves classical effectors of apoptosis or immune signaling, prior studies indicate that RNA-binding and splicing regulators such as **YBX1** and **TIA1** may modulate downstream processes by influencing transcript stability or alternative splicing [59, 60]. A recent study by **Hessman et al.** reported a novel role of extracellular YB1, which, together with progranulin, interferes with the binding of tumor necrosis factor (TNF) to its receptor TNFR1 [61]. Given that TNF regulates diverse cellular processes including inflammation, cell proliferation, differentiation, and apoptosis, this observation suggests a potential immunomodulatory role of YBX1. In addition, **YBX1 and TIA1** have been implicated in **Fas receptor alternative splicing**, which is directly relevant to apoptotic signaling [62, 63]. **CFH**, as a key complement regulator, may also impact immune responses via complement activation pathways [64].

To validate these enrichment results, we conducted supplementary differential expression analyses of representative pathway components (Supplementary Table 5). Chemokines such as C-C Motif Chemokine Ligand 2 (CCL2), C-C Motif Chemokine Ligand 5 (CCL5), C-X-C Motif Chemokine Ligand 12 (CXCL12), and C-X-C Motif Chemokine Ligand 18 (CXCL8) were significantly upregulated, while CCR2 was markedly downregulated, a pattern consistent with enhanced monocyte trafficking and a microenvironment favorable for M2 macrophage polarization. ECM-related genes including Hyaluronan Synthase 2 (HAS2), Versican (VCAN), Collagen Type I Alpha 1 Chain (COL1A1), Collagen Type III Alpha 1 Chain (COL3A1), Collagen Type V Alpha 1 Chain (COL5A1), together with Transforming Growth Factor Beta 3 (TGFB3), were strongly upregulated, consistent with active fibrotic remodeling. In apoptotic signaling, Caspase 3 (CASP3), Fas Cell Surface Death Receptor (FAS), and TNF Receptor Superfamily Member 10B (TNFRSF10B) were significantly increased, indicating activation of executioner caspases.

These observations suggest that, although the seven feature genes are not direct effectors of these pathways, they may function within regulatory networks that intersect with key processes such as epithelial survival, immune modulation, and fibrotic remodeling, thereby potentially contributing to IPF pathogenesis.

### Subtype classification of ipf based on feature gene signatures

**Fig. 3:**
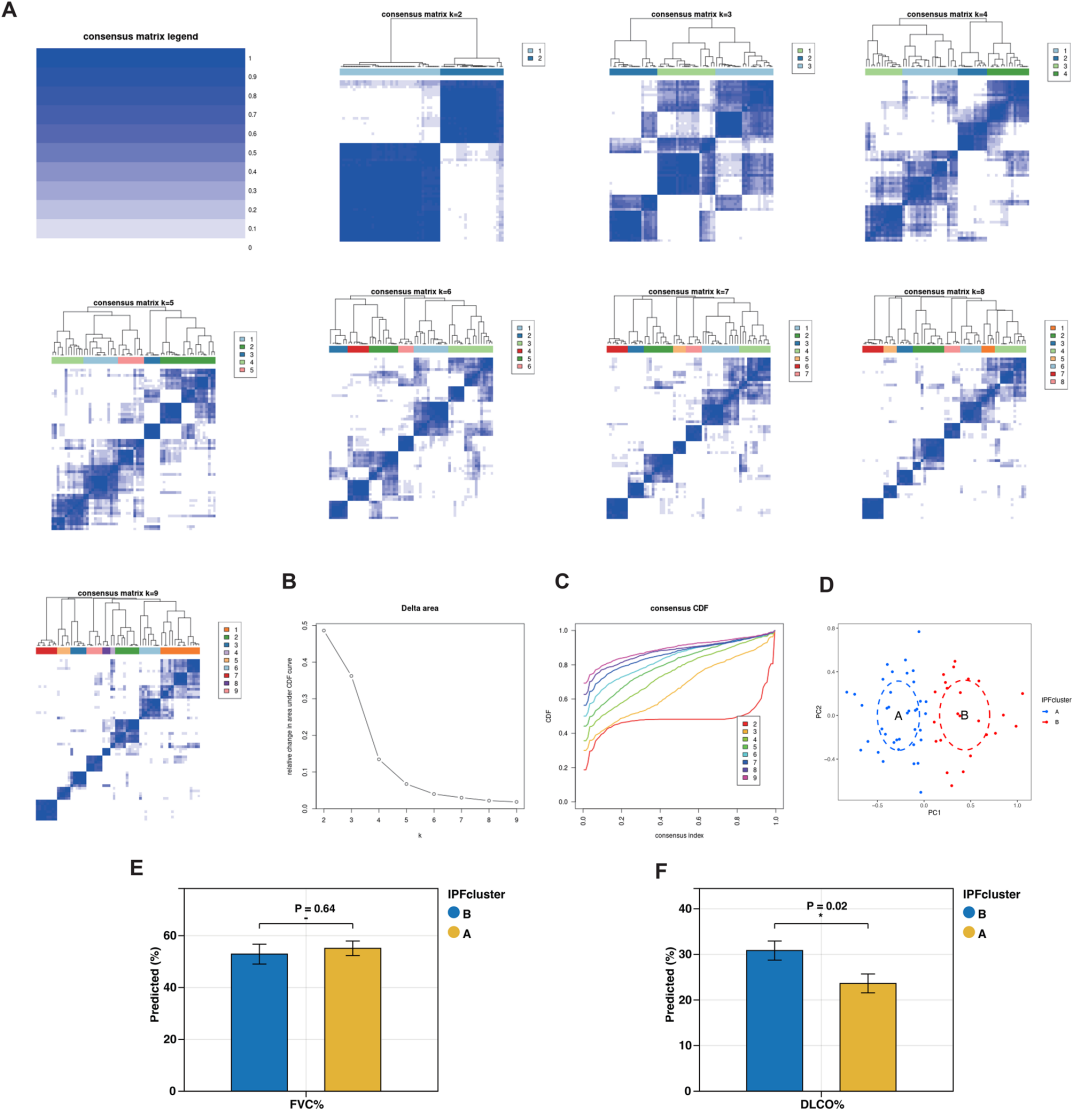
The results of 2 clusters of patients with IPF. **A**: Heatmap of consensus clustering for k = 2. Color gradients indicate from 0 to 1 (white: 0, blue: 1). **B**: Delta represents the relative change course in the area under the CDF curve when k = 2–9. **C**: Cumulative distribution function (CDF); **D**: PCA results of the expression profiles of the two IP cluster patterns, showing the marked differences in the transcriptomes between the different IPFcluster. The red dots in the scatter plot represent IPFcluster A, and the blue dots represent IPFcluster B. **E**: Comparison of predicted forced vital capacity percentage (FVC%) between Cluster A and Cluster B using unpaired two-tailed t-tests. **F**: Comparison of predicted diffusion capacity of carbon monoxide percentage (DLCO%) between Cluster A and Cluster B using unpaired two-tailed t-tests

Using the identified feature gene set, we performed consensus clustering to stratify IPF samples and identified two distinct molecular subtypes (Fig. 3A). The optimal number of clusters was determined through the combined evaluation of the consensus matrix heatmap, empirical cumulative distribution function (CDF) plot, and delta area plot. The results indicated that clustering stability peaked when *k* = 2 (Fig. 3B). Moreover, a notable change in the relative area under the CDF curve was observed between *k* = 2 and *k* = 9 (Fig. 3C). Principal component analysis (PCA) further confirmed a clear separation between IPF subtypes A and B (Fig. 3D). In addition, we calculated a RiskScore for each IPF sample (Supplementary Table 3).

To further elucidate the clinical relevance of our classification results, we compared the pulmonary function parameters between the two IPF subtypes. Specifically, the predicted forced vital capacity percentage (FVC%) and predicted diffusion capacity of carbon monoxide percentage (DLCO%) were assessed. The analysis revealed that patients in Cluster A exhibited slightly higher FVC% values than those in Cluster B; however, the difference did not reach statistical significance (Fig. 3E, *p* = 0.15). In contrast, DLCO% was significantly lower in Cluster A compared to Cluster B (Fig. 3F, *p* = 0.02), indicating more pronounced impairment of gas exchange capacity in this subgroup. These findings suggest that the proposed classification captures clinically meaningful heterogeneity, with Cluster A potentially representing a subgroup of patients experiencing more severe functional decline.

Nevertheless, we acknowledge certain limitations. The observed difference in FVC% between the two clusters did not reach statistical significance, and treatment-response or long-term survival data were not available in the analyzed cohorts. Therefore, while our subtype classification shows clear molecular and functional distinctions, further validation in prospective clinical studies with detailed outcome data is needed to establish its full prognostic and therapeutic relevance.

### Immune microenvironment alterations and their clinical relevance in IPF

**Fig. 4:**
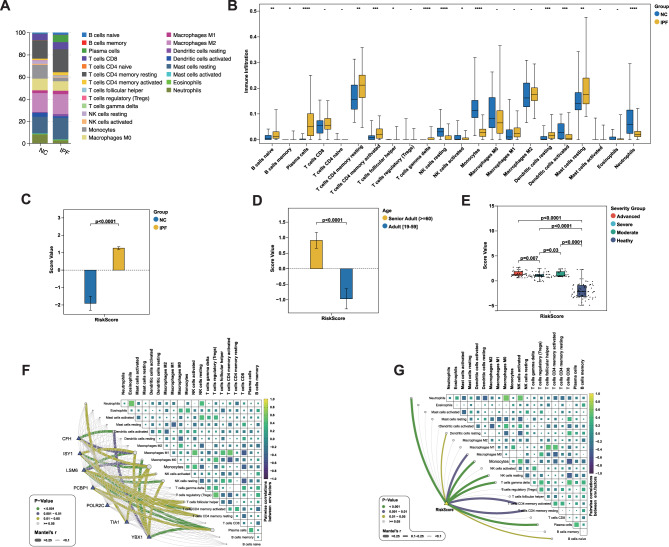
**A–B**: Comparison of immune cell infiltration levels between idiopathic pulmonary fibrosis (IPF) patients and normal controls (NC). Statistical significance was determined using unpaired two-tailed t-tests. **C**: Comparison of RiskScore between NC (blue) and IPF (yellow) patients. The IPF group showed a significantly higher RiskScore compared to the NC group (p < 0.0001, unpaired two-tailed t-tests). **D**: RiskScore comparison between different age groups, showing a significant difference between senior adults (yellow, age ≥ 60) and adults (blue, age 19–59), with the senior group having higher RiskScore (p < 0.001, unpaired two-tailed t-tests). **E**: Comparison of RiskScore based on disease severity. RiskScore increased significantly with greater disease severity (p < 0.0001, one-way ANOVA). Severity groups include healthy (purple), moderate (green), severe (sky blue), and advanced (red). **F**: Correlation network between seven feature genes and immune cells. Line colors indicate statistical significance: green (p < 0.001), purple (0.001 ≤ p < 0.01), yellow (0.01 ≤ p < 0.05), and grey (p ≥ 0.05). Correlations were assessed using Pearson’s correlation analysis. **G**: Correlation analysis between immune cell subpopulations and RiskScore. Color intensity indicates the strength of correlation, and line colors and thickness represent the magnitude and significance of the correlations (Pearson’s method, p-values). *p < 0.05; **P < 0.01; ****P < 0.001

To explore the immunological landscape of IPF, we employed the CIBERSORT algorithm with the LM22 reference matrix to deconvolute bulk RNA-seq profiles and estimate the proportions of 22 immune cell subsets (Supplementary Table 4). This approach allows robust inference of immune cell fractions, including M0, M1, and M2 macrophages, based on predefined transcriptomic signatures rather than single surface markers. As illustrated (Fig. 4A–B, Supplementary Table 5–2), IPF tissues exhibited elevated proportions of naïve B cells, memory B cells, plasma cells, resting CD4 memory T cells, activated CD4 memory T cells, follicular helper T cells, gamma delta T cells, resting dendritic cells, and resting mast cells. Conversely, resting NK cells, activated dendritic cells, and neutrophils were decreased in IPF compared with normal controls. The observed reduction in neutrophils may reflect the transition from acute neutrophil-dominated inflammation to macrophage- and lymphocyte-driven chronic remodeling characteristic of IPF. Demographic analysis further indicated that IPF patients were significantly older than non-diseased controls (mean 63 ± 7 vs. 48 ± 15 years, *p* = 0.004), which may also contribute to immune profile differences. In addition, because the transcriptomic data were obtained from publicly available GEO cohorts, detailed clinical metadata such as occupational or environmental exposure histories were not available. Thus, while our findings highlight clear immunological differences between groups, we acknowledge that unmeasured confounders, including environmental and occupational factors, may also have influenced the observed immune landscape.

To further dissect the relationship between the seven selected feature genes and immune cell infiltration in IPF, we conducted a correlation analysis. As shown in Fig. 4F, **YBX1** expression was positively correlated with M0 and M1 macrophages, monocytes, and resting NK cells, but negatively correlated with activated dendritic cells and plasma cells. **CFH** exhibited a similar correlation pattern. **LSM6** and **ISY1** demonstrated strong positive correlations with M1 macrophages, monocytes, resting NK cells, and both resting and activated CD4 memory T cells, while negatively correlating with plasma cells. In contrast, **POLR2C** and **PCBP1** showed weaker associations with immune cell infiltration (Supplementary Table 5–3). These findings suggest that the feature genes may influence the recruitment and activation of immune cells in the pulmonary microenvironment, potentially contributing to IPF pathogenesis via immune modulation.

Furthermore, we introduced a novel scoring system—**RiskScore**—derived from PCA, designed to capture the underlying genetic and molecular complexity of IPF. Using bulk RNA-seq datasets, we systematically evaluated the association between the RiskScore and various clinical features of IPF to assess its prognostic relevance and clinical utility. As depicted in the analysis, the RiskScore effectively distinguished IPF patients from healthy controls (Fig. 4C) and demonstrated significant stratification across age groups (Fig. 4D) and disease severity (Fig. 4E). We also investigated its relationship with immune cell populations (Fig. 4 G, Supplementary Table 5–4), highlighting its potential involvement in the immunopathogenesis of IPF. Collectively, these results underscore the RiskScore as a robust tool for differentiating disease states, assessing disease burden, and potentially guiding clinical decision-making in IPF management.

### Remodeling of AT2–macrophage crosstalk via C3 signaling in IPF

Single-cell RNA sequencing (scRNA-seq) analysis revealed distinct differences in cellular composition (Fig. S1A) and gene expression profiles (Fig. S1C) between NC and patients with IPF. As shown in Fig. S1B, the relative proportions of various cell types were significantly altered in the IPF group. Notably, macrophage populations exhibited pronounced shifts, particularly in M0 and M2 subtypes, likely reflecting the inflammation commonly associated with fibrotic progression. Fibroblasts were markedly enriched in IPF samples, consistent with their established role in extracellular matrix production and tissue remodeling. A substantial reduction in alveolar type II (AT2) cells was also observed, highlighting impaired epithelial integrity.

Given the pivotal role of AT2 cells in maintaining alveolar homeostasis, their loss raised the possibility that surviving AT2 subpopulations may undergo functional reprogramming and actively reshape the local immune environment. To test this hypothesis, we applied CellChat analysis to the scRNA-seq data to map intercellular communication networks, with a particular focus on epithelial–macrophage interactions.

**Fig. 5:**
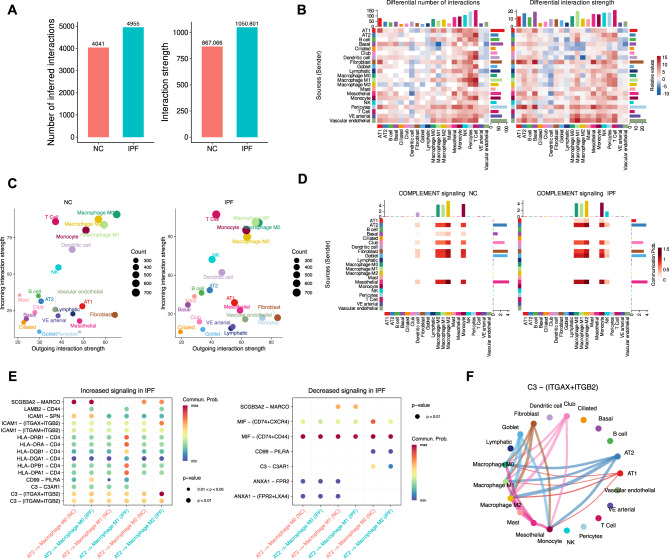
**A**: Comparison of the number of cell-cell interactions and their interaction strength between NC and IPF samples. **B**: Heatmap showing differences in cell-cell communication between NC and IPF samples, including the number of interactions and interaction strength, stratified by cell type. **C**: Bubble plot depicting the signaling strength of incoming (received) and outgoing (sent) signals across different cell types in NC and IPF samples. **D**: Differential activity of the complement signaling pathway in cell-cell communication between the two groups. **E**: Altered C3 signaling pathway in IPF, particularly in interactions among macrophages, AT2 cells, and epithelial cells. **F**: Changes in the ligand-receptor pair ITGAX+ITGB2 in IPF, highlighting enhanced interactions between macrophages and AT2 cells

The results revealed striking remodeling of cell–cell communication in IPF. Both the number and strength of signaling interactions were markedly increased (Fig. 5A–B), forming a denser and more intricate communication topology compared with NC. Among immune lineages, macrophage-mediated signaling showed the most pronounced enhancement (Fig. 5C), suggesting that macrophages play a central role in amplifying local immune responses and fibrosis.

Of particular interest, complement signaling was strongly upregulated in IPF, prominently facilitating interactions between AT2 cells and macrophages (Fig. 5D). Given the critical role of AT2 cells in alveolar repair, enhanced crosstalk with immune cells likely exacerbates inflammation and promotes fibrosis. Among complement pathways, the C3 signaling axis was significantly activated in IPF (Fig. 5E), with increased activity observed in interactions among macrophages, AT2 cells, and other epithelial cell types. These findings suggest that the complement system may function as a key modulator of immune responses and fibrotic development in IPF.

Further analysis identified specific signaling interactions mediated by C3 receptors—namely ITGAX and ITGB2—between AT2 cells and the M2 macrophage subpopulation (Fig. 5F). This interaction implies that AT2 cells may directly engage M2 macrophages through C3-dependent signaling, potentially accelerating fibrotic progression. These results highlight a previously underappreciated role for AT2 cells in coordinating immune cell recruitment and in amplifying fibrosis through specific ligand–receptor pathways, with important implications for therapeutic targeting.

### Validation of alveolar macrophage remodeling in a bleomycin-induced mouse Model

To complement our findings from human IPF lungs, we performed an in-depth analysis of publicly available single-cell RNA sequencing (scRNA-seq) data from a bleomycin (BLM)-induced pulmonary fibrosis mouse model (GSE240134). This dataset included wild-type (WT), day 14 (BLM14), and day 28 (BLM28) post-treatment groups, providing a dynamic view of alveolar macrophage (AM) remodeling during fibrotic progression.

**Fig. 6:**
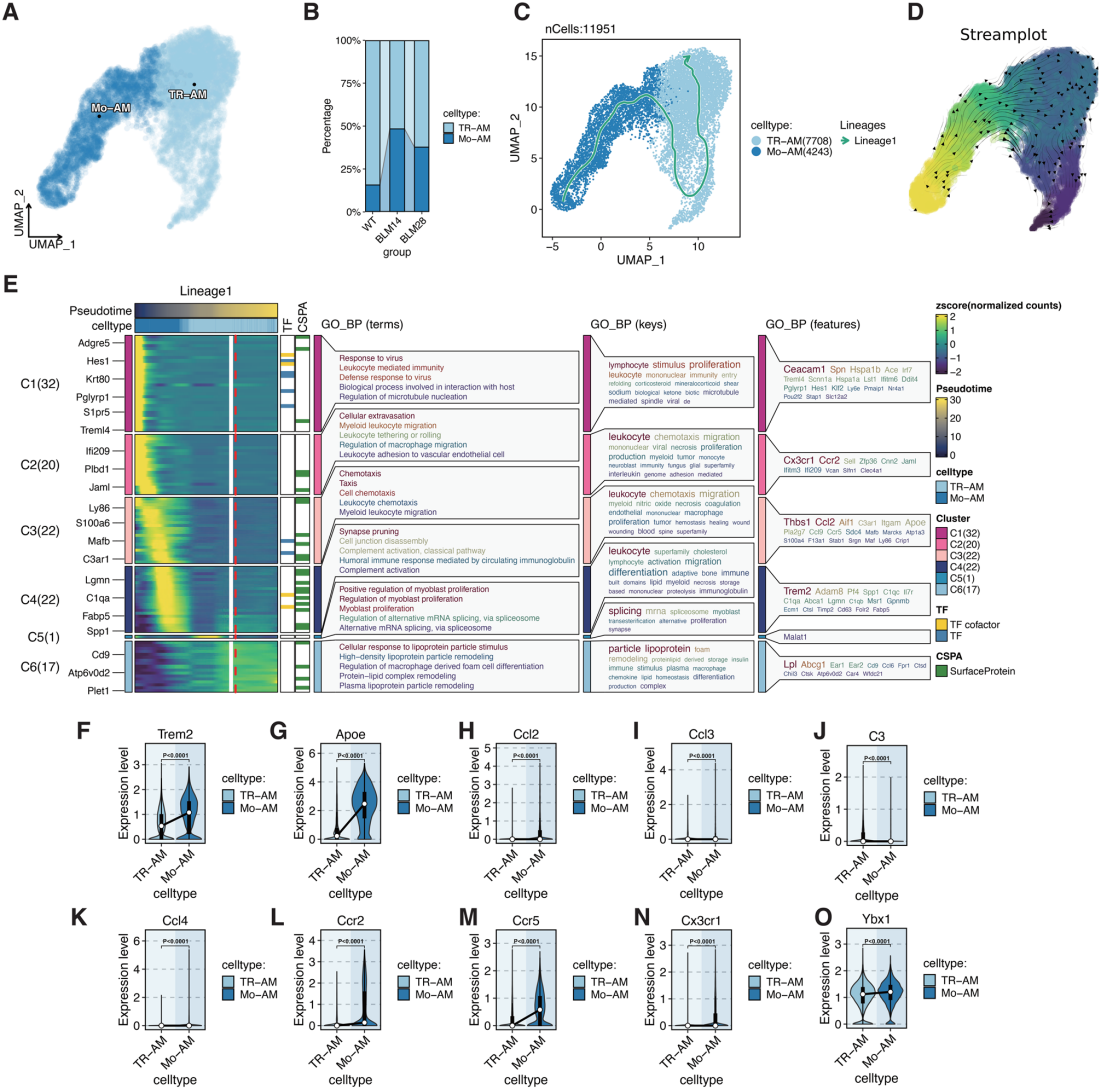
SnRNA-seq analysis of AMs from a BLM-induced pulmonary fibrosis mouse model. **A**: UMAP visualization of AMs identifies two major subpopulations: tissue-resident AMs (TR-AMs) and monocyte-derived AMs (mo-AMs). **B**: proportional changes in TR-AMs and Mo-AMs across WT, BLM14, and BLM28 groups. Mo-AMs markedly expanded at BLM14, accompanied by a significant reduction in TR-AMs, with partial recovery of TR-AMs at BLM28. C–D: trajectory analysis using slingshot (**C**) and StaVIA (**D**) demonstrates a differentiation trajectory from Mo-AMs toward TR-AMs, indicating dynamic macrophage plasticity during fibrotic progression. **E**: heatmap of gene dynamics along slingshot lineage 1 shows progressive downregulation of Trem2, apoe, Ccl2, Ccl3, Ccl4, Ccr2, Ccr5, and Cx3cr1 during the Mo-AM to TR-AM transition. GO enrichment analysis highlights biological processes related to leukocyte migration, chemotaxis, viral defense, and complement activation. F–O: differential expression analyses of representative genes between TR-AMs and Mo-AMs. Trem2, apoe, Ccl2, Ccl3, Ccl4, Ccr2, Ccr5, Cx3cr1, and Ybx1 were preferentially expressed in Mo-AMs, whereas *C3* was selectively upregulated in TR-AMs

Unsupervised clustering identified two major AM subsets: tissue-resident AMs (TR-AMs) and monocyte-derived AMs (Mo-AMs) (Fig. 6A). Quantitative analysis demonstrated that Mo-AMs markedly expanded at BLM14, coinciding with a significant reduction in TR-AMs. By BLM28, TR-AMs exhibited partial recovery, whereas Mo-AMs remained elevated (Fig. 6B). Trajectory analysis using Slingshot (Fig. 6C) and StaVia (Fig. 6D) consistently indicated a differentiation trajectory from Mo-AMs toward TR-AMs, highlighting the dynamic plasticity of AM subsets in response to fibrotic injury.

Lineage analysis (Fig. 6E) revealed that a cluster of inflammatory and chemotactic genes—including Trem2, Apoe, Ccl2, Ccl3, Ccl4, Ccr2, Ccr5, and Cx3cr1—were predominantly expressed in Mo-AMs and showed a progressive decline along the Mo-AM–to–TR-AM trajectory. Gene Ontology enrichment indicated that these genes were enriched in biological processes such as viral response, leukocyte-mediated immunity, macrophage migration, chemotaxis, and complement activation, suggesting that Mo-AMs at early fibrotic stages orchestrate leukocyte recruitment and inflammatory amplification.

A particularly notable finding was the distinct and reciprocal pattern of Ybx1 and C3 expression observed in TR-AMs (Fig. 6F–O). Specifically, TR-AMs exhibited significant downregulation of Ybx1 accompanied by marked upregulation of C3. This pattern mirrors our observations in human scRNA-seq data, where YBX1 downregulation coincided with increased C3 expression in fibrotic lungs, supporting a conserved YBX1–C3 regulatory axis across species. In contrast, Mo-AMs were characterized by elevated levels of chemokines such as Ccl2, Ccl3, and Ccl4, highlighting their role in immune cell recruitment and early inflammatory amplification. Together, these findings suggest that while Mo-AMs contribute to the inflammatory milieu through chemokine signaling, TR-AMs may sustain complement activation via the YBX1–C3 axis, thereby perpetuating immune–epithelial crosstalk and fibrotic remodeling.

### Identification and trajectory inference of AT2 cell subtypes

**Fig. 7:**
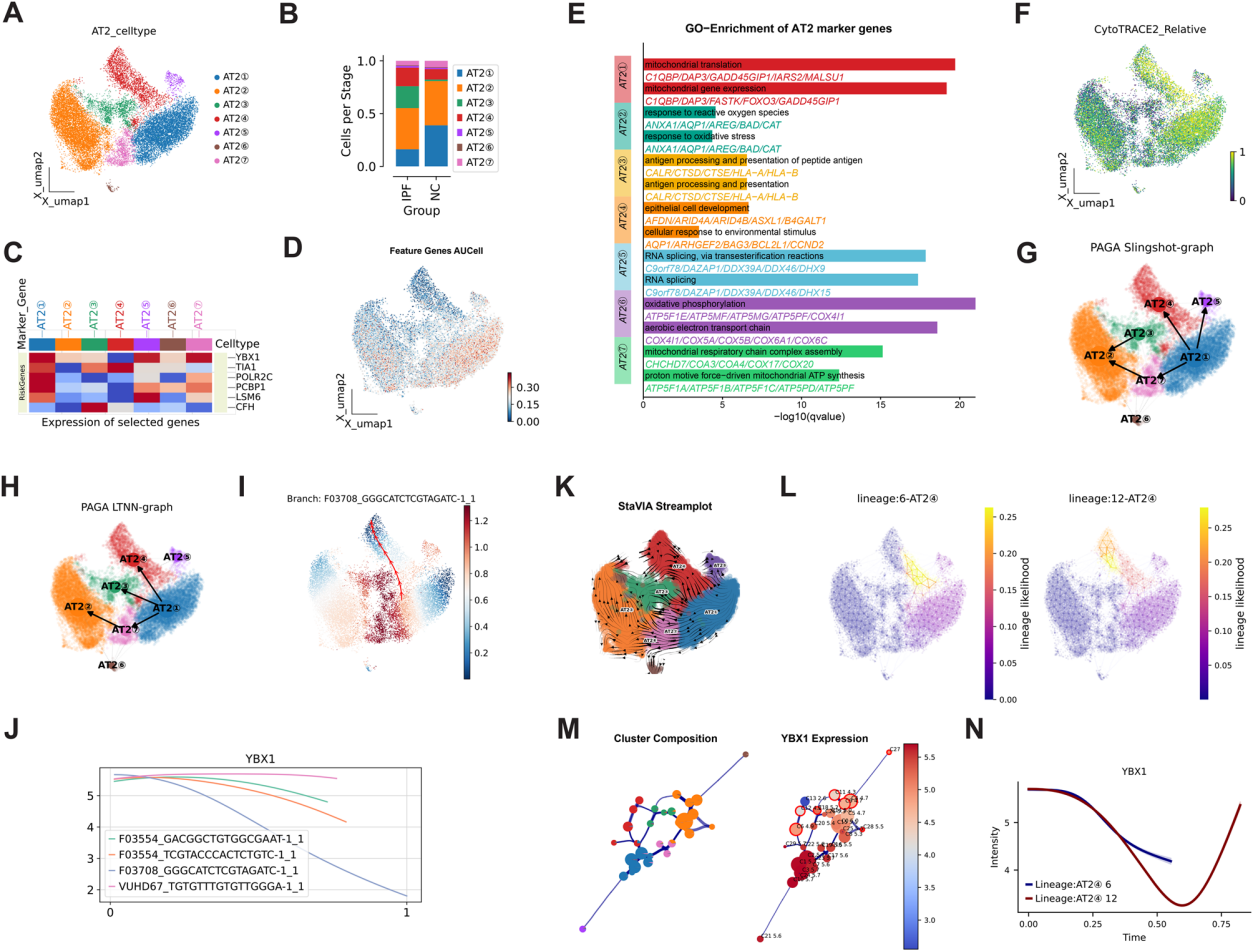
**A**: UMAP projection of AT2 cell subtypes (AT2① to AT2⑦) based on their gene expression profiles. Each subtype is represented by a different color, showing clear separation in the UMAP space. **B**: Bar plot showing the relative proportions of AT2 subtypes in the IPF and NC groups. **D**: The UMAP heatmap overlay of feature genes AUCell scores across the AT2 cell population shows higher AUCell scores in certain AT2 subtypes, particularly AT2① and AT2②. **E**: Gene ontology (GO) enrichment analysis of AT2 marker genes. Each bar represents enriched biological processes for a specific AT2 subtype. The length of the bar indicates the level of significance (−log10(q-value)). **F**: CytoTRACE analysis showing cellular differentiation potential. **G-H**: Cell trajectories and network topology inferred through Slingshot/Palantir and PAGA algorithms. Arrows indicate potential pathways of differentiation, illustrating the interconnected nature of cell states and their evolution during lung tissue development and disease progression. I-J: Palantir-based identification of key developmental branches (**I**) and dynamic changes in YBX1 expression (**J**). **K–N**: StaVIA reveals developmental branching structure (**K**), identifies key branch points (**L**), and delineates YBX1 expression dynamics across distinct trajectories (M–N)

To investigate the heterogeneity and potential functional roles of AT2 cells in IPF, we performed subtype classification and identified seven distinct AT2 subpopulations (AT2① through AT2⑦) (Fig. 7A). The proportions of these subtypes varied markedly between NC and IPF samples, suggesting disease-specific cellular reprogramming. Notably, AT2③ and AT2④ were significantly enriched in IPF tissues (Fig. 7B), indicating their potential involvement in disease pathogenesis.

We next examined the expression of the previously defined feature gene set across these AT2 subtypes (Fig. 7C) and evaluated their transcriptional activity using AUCell scoring (Fig. 7D). The gene set exhibited high activity in AT2① and AT2②, but was largely suppressed in AT2③ and AT2④, implying a crucial role in maintaining normal cellular function. Gene Ontology (GO) enrichment analysis revealed that AT2① and AT2② were highly enriched in metabolic pathways, particularly mitochondrial translation and gene expression, suggesting they represent metabolically active populations essential for alveolar homeostasis. AT2⑥ and AT2⑦ were enriched in oxidative phosphorylation and mitochondrial respiratory chain assembly pathways, indicating a key role in energy metabolism and mitochondrial function. In contrast, AT2⑤ showed significant enrichment in RNA processing, splicing, and metabolic pathways, implying a potential regulatory role in post-transcriptional RNA modification and cellular stress responses within the fibrotic microenvironment.

AT2③ cells were associated with oxidative stress response and antigen processing and presentation pathways, indicating activation of immune functions and involvement in modulating the local inflammatory milieu. AT2④ cells were enriched in pathways related to epithelial development and environmental response, including adaptation to oxidative stress, pathogen exposure, and tissue injury, suggesting their identity as reactive AT2 subtypes induced by disease stimuli (Fig. 7E).

Developmental state analysis using CytoTRACE revealed that AT2① had the highest differentiation potential, suggesting it represents a progenitor-like population (Fig. 7F). We employed the Slingshot algorithm to reconstruct the differentiation trajectory of AT2 cells, identifying multiple lineage branches originating from AT2① and progressing toward other subtypes, including AT2② and AT2④ (Fig7 G). This was further validated by the Palantir algorithm, which refined pseudotime inference and delineated representative differentiation branches (e.g., F03708_GGGCACTCCTGAGATC), revealing continuous transitions in cellular states. Notably, the transcriptional regulator **YBX1** exhibited a dynamic, time-dependent expression pattern along the pseudotime trajectory (Fig. 7I–J), suggesting a potential regulatory role in fate determination.

To further integrate lineage transitions with cellular fate decisions, we applied the StaVIA algorithm to visualize differentiation flow dynamics. This analysis revealed two principal trajectories: one toward AT2② and another toward AT2④ (Fig. 7K). Among them, lineage branches 6 and 12 were representative paths leading to AT2④ differentiation (Fig. 7 L). Spatial clustering patterns (left panel, Fig. 5 M) and **YBX1** expression gradients (right panel, Fig. 7 M) jointly demonstrated spatial heterogeneity in **YBX1** expression. Along both pseudotime trajectories, **YBX1** expression exhibited a biphasic trend—initially decreasing before rising again (Fig. 7N)—suggesting its involvement in early-stage regulation of AT2 fate decisions and subtype specification.

In summary, our study delineates the extensive heterogeneity and functional diversification of AT2 cells in IPF. The observed shifts in developmental trajectories and transcriptional reprogramming highlight the dynamic plasticity of these cells under fibrotic stress. **YBX1** emerges as a potential key regulator orchestrating the transition from progenitor to functionally distinct AT2 subtypes. These findings offer novel insights into the mechanisms underlying epithelial injury, aberrant repair, and alveolar regeneration in IPF, and identify promising targets for therapeutic intervention.

### Mendelian randomization analysis reveals a causal role of YBX1 in pulmonary function

To explore the potential causal relationship between **YBX1** and lung function, we performed Mendelian randomization (MR) analyses using forced expiratory volume in one second (FEV₁) and forced vital capacity (FVC) as outcome traits. These analyses aimed to elucidate the role of **YBX1** in the functional differentiation of AT2 cells, particularly within the pathophysiological context of pulmonary fibrosis (see Supplementary Table 6).

**Fig. 8:**
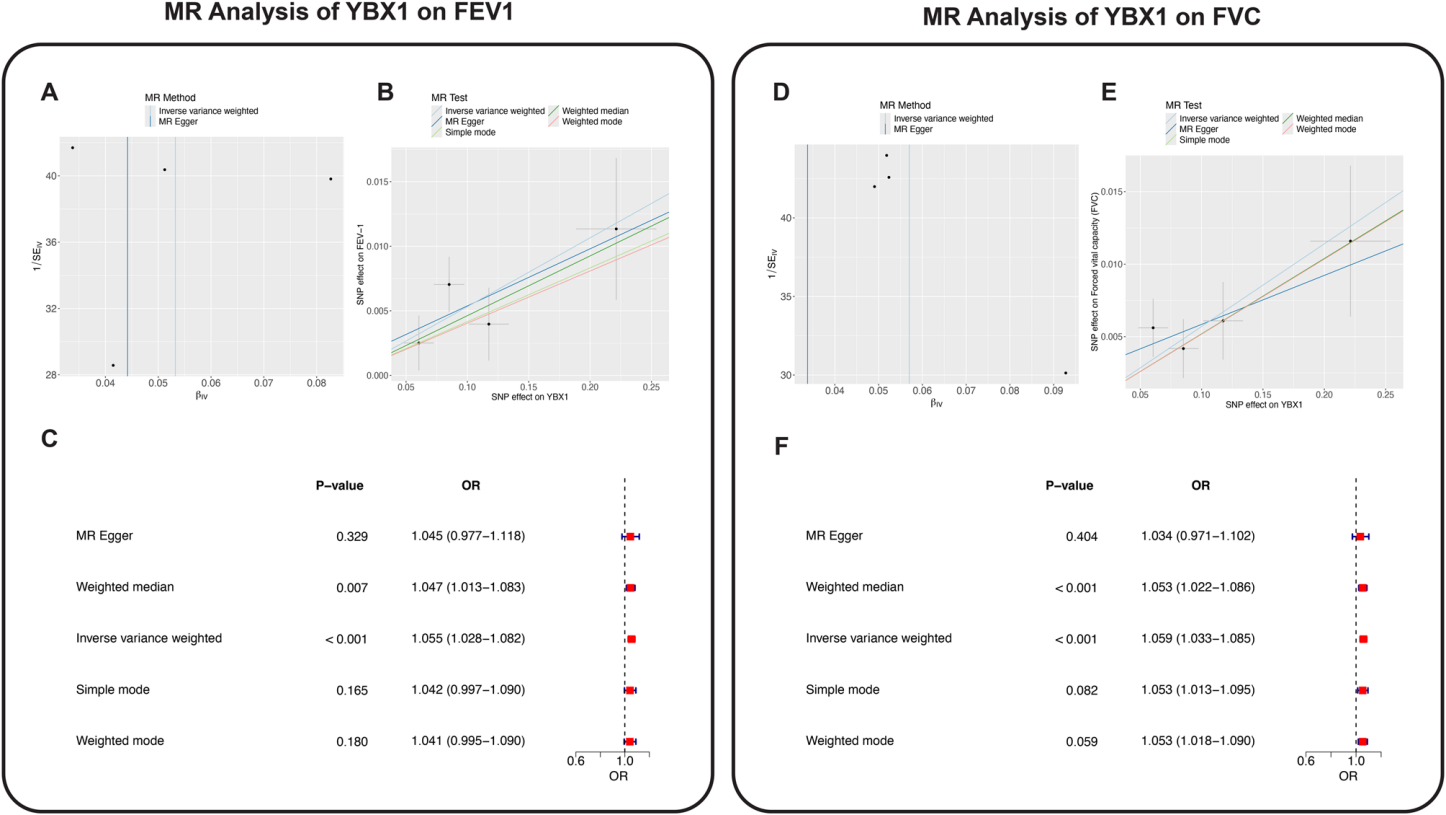
**A:** IVW regression analysis of YBX1 and FEV1, showing the causal effect of YBX1 on FEV1. **B:** MR analysis of YBX1 and FEV1, presenting the regression estimates from IVW, MR Egger, Simple Mode, and Weighted Mode methods. **C:** Causal effect estimates for YBX1 on FEV1 across different MR methods, including Odds Ratios (OR) and their 95% confidence intervals. **D:** IVW regression analysis of YBX1 and FVC, showing the causal effect of YBX1 on FVC. **E:** MR analysis of YBX1 and FVC, presenting the regression estimates from IVW, MR Egger, Simple Mode, and Weighted Mode methods. **F:** Causal effect estimates for YBX1 on FVC across different MR methods, including Odds Ratios (OR) and their 95% confidence intervals

For FEV₁, MR analysis indicated a positive association with **YBX1** expression (Fig. 8A–C). The inverse-variance weighted (IVW) method estimated an odds ratio (OR) of 1.055 (95% CI: 1.028–1.082), suggesting that increased **YBX1** expression is associated with improved lung function. The MR-Egger method yielded an OR of 1.045 (95% CI: 0.977–1.118), consistent with the null hypothesis, yet directionally aligned with IVW estimates. The weighted median method showed a statistically significant association (OR = 1.047, *p* = 0.007), reinforcing the potential beneficial effect of **YBX1** on pulmonary function.

Similarly, MR analysis of FVC supported a positive causal relationship with **YBX1** expression (Fig. 8D–F). The IVW method yielded an OR of 1.059 (95% CI: 1.033–1.085), indicating a putative protective effect on overall lung capacity. Although the MR-Egger estimate did not reach statistical significance (OR = 1.034, 95% CI: 0.971–1.102), the weighted median approach revealed a robust association (OR = 1.053, *p* < 0.001), further substantiating the role of **YBX1** in maintaining pulmonary function.

To assess the robustness of these findings, we conducted tests for horizontal pleiotropy and heterogeneity. The MR-Egger intercepts for FEV₁ and FVC were not statistically significant (*p* = 0.56 and *p* = 0.82, respectively), indicating minimal influence from horizontal pleiotropy. Additionally, heterogeneity tests across all MR methods revealed no significant inconsistencies (*p* > 0.05), supporting the stability and reliability of our results.

Integrating these genetic findings with the observed functional heterogeneity and trajectory dynamics of AT2 cells in IPF, we propose that **YBX1** functions as a key developmental regulator. It may guide AT2 cells from a progenitor state toward disease-associated functional subtypes via transcriptional reprogramming. This regulatory role of **YBX1** is likely critical for orchestrating epithelial repair and regeneration under pathological conditions. Collectively, our MR analysis provides genetic evidence supporting a causal role for **YBX1** in pulmonary function, with implications for its potential as a therapeutic target in fibrotic lung disease.

### Multicenter data and in vitro experiments reveal YBX1 expression patterns in IPF

In this study, we identified a potential role for YBX1 in the cellular stress response and injury repair of AT2 cells, particularly under pathological conditions of IPF. To verify this hypothesis, we employed both multicenter transcriptomic analyses and in vitro experiments to validate changes in YBX1 expression associated with IPF.

**Fig. 9:**
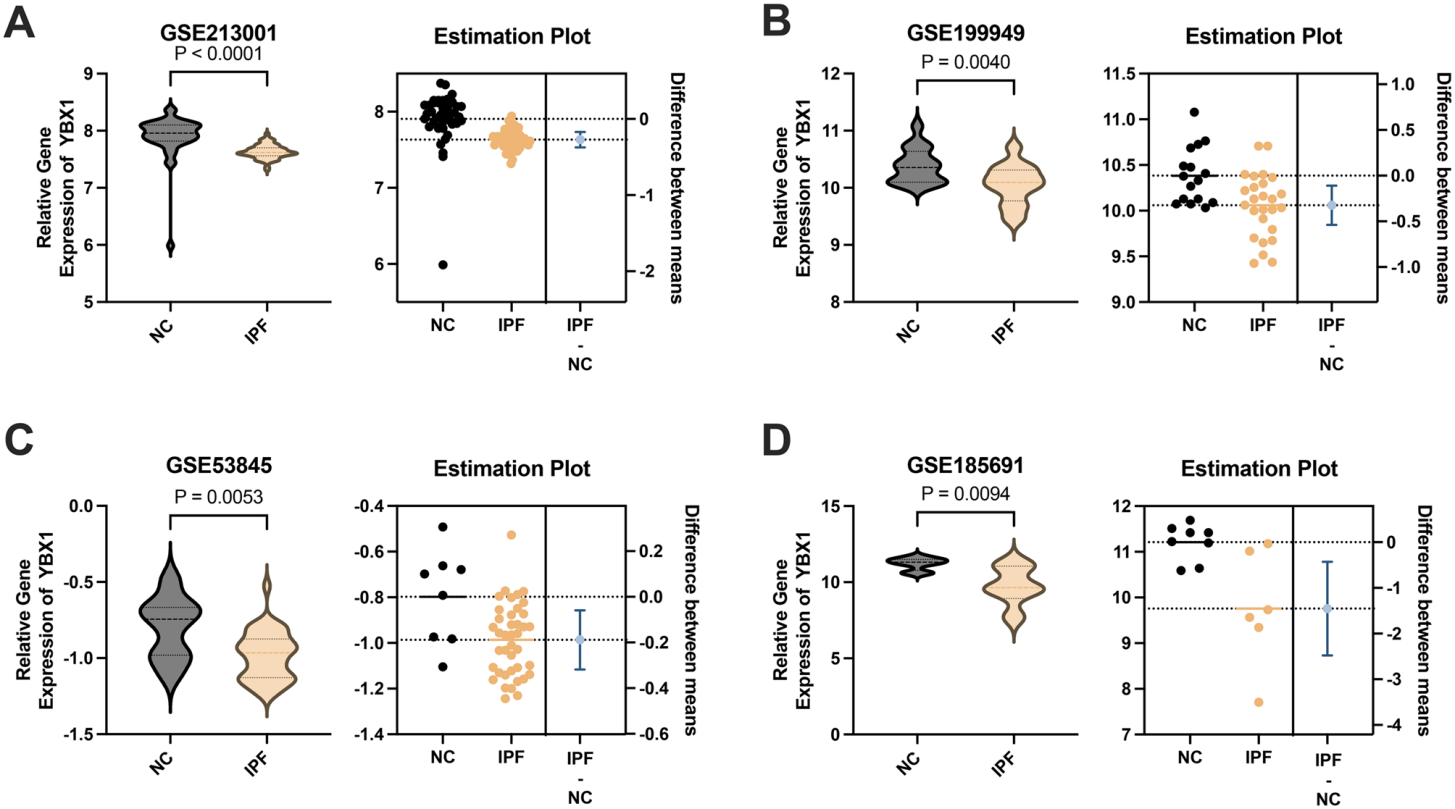
**A–D**: Violin plots (left panels) depict the distribution of YBX1 gene expression levels in lung tissue samples from IPF patients and NC in four independent datasets: (**A**) GSE213001, (**B**) GSE199949, (**C**) GSE53845, and (**D**) GSE185691. Corresponding estimation plots (right panels) illustrate the mean differences in YBX1 expression between IPF and NC groups, with each dot representing an individual sample. Horizontal bars indicate group means, and vertical error bars represent 95% confidence intervals of the mean difference. A consistent and statistically significant downregulation of YBX1 was observed in IPF samples across all datasets. Statistical significance was assessed using unpaired two-tailed t-tests

Multicenter Data Analysis (Fig. 9): To assess YBX1 expression in IPF, we integrated and analyzed four independent publicly available transcriptomic datasets (GSE213001, GSE199949, GSE53845, and GSE185691). Across all datasets, YBX1 expression was significantly downregulated in lung tissues from IPF patients compared to NC. In GSE213001, YBX1 expression showed the most pronounced decrease (*p* < 0.0001). Similar trends were observed in the other three datasets, all reaching statistical significance (*p* < 0.01). Forest plots of effect size and confidence intervals further confirmed the mean differences in YBX1 expression between IPF and NC samples across datasets, suggesting a consistent biological role for YBX1 in IPF. The reproducibility of this result across multiple independent cohorts underscores the potential of YBX1 as a candidate biomarker or therapeutic target.

**Fig. 10:**
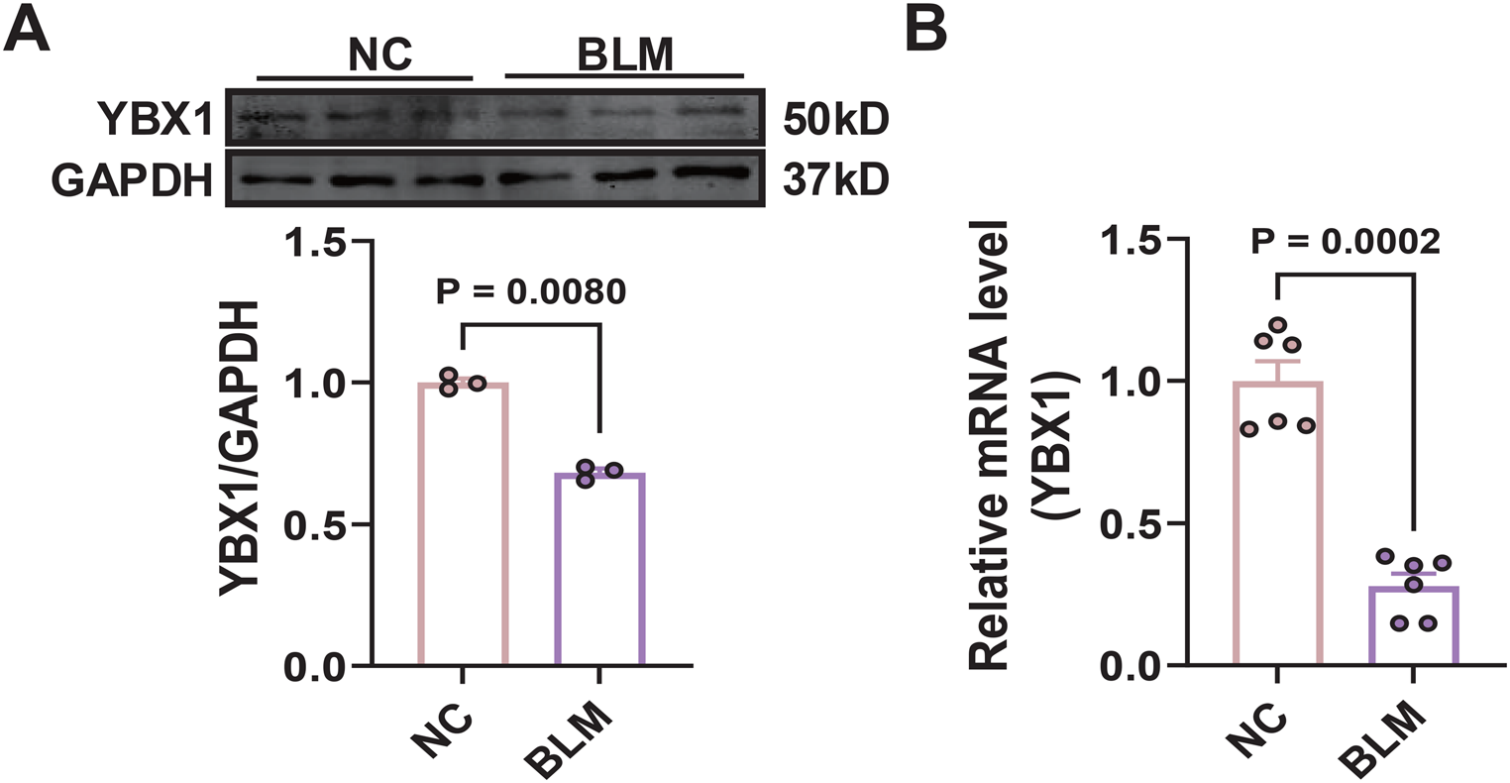
**A**: Western blot analysis showing YBX1 protein levels in normal control (NC) and bleomycin-treated (BLM) cells, with GAPDH used as a loading control. **B**: Quantitative PCR results displaying the relative mRNA levels of YBX1 in NC and BLM groups

In Vitro Validation: Western blot analysis (Fig. 10A) demonstrated a marked reduction in YBX1 protein levels in bleomycin-treated MLE-12 cells, with GAPDH serving as the loading control. Quantitative analysis confirmed this reduction, clearly indicating the suppressive effect of bleomycin on YBX1 protein expression. Complementarily, qPCR results (Fig. 10B) showed a parallel decrease in YBX1 mRNA levels in bleomycin-treated cells, further supporting the conclusion that YBX1 is significantly downregulated in fibrotic conditions.

In summary, the integration of multicenter transcriptomic analysis and in vitro cellular modeling provides robust evidence for the downregulation of YBX1 in IPF. This consistent expression pattern—observed in both patient lung tissues and fibrotic injury models—strongly supports a potential regulatory role for YBX1 in IPF pathogenesis and highlights its value as a prospective diagnostic biomarker or therapeutic target.

### YBX1 enhances oxidative stress tolerance in AT2 cells by regulating mitochondrial function

Through a series of mechanistic experiments, this study elucidates the critical role of YBX1 under bleomycin-induced oxidative stress, particularly in alveolar type II (AT2) cells (MLE-12). The data reveal that YBX1 significantly influences mitochondrial health and metabolic activity, offering new mechanistic insights into its function in cellular homeostasis and injury adaptation.

**Fig. 11:**
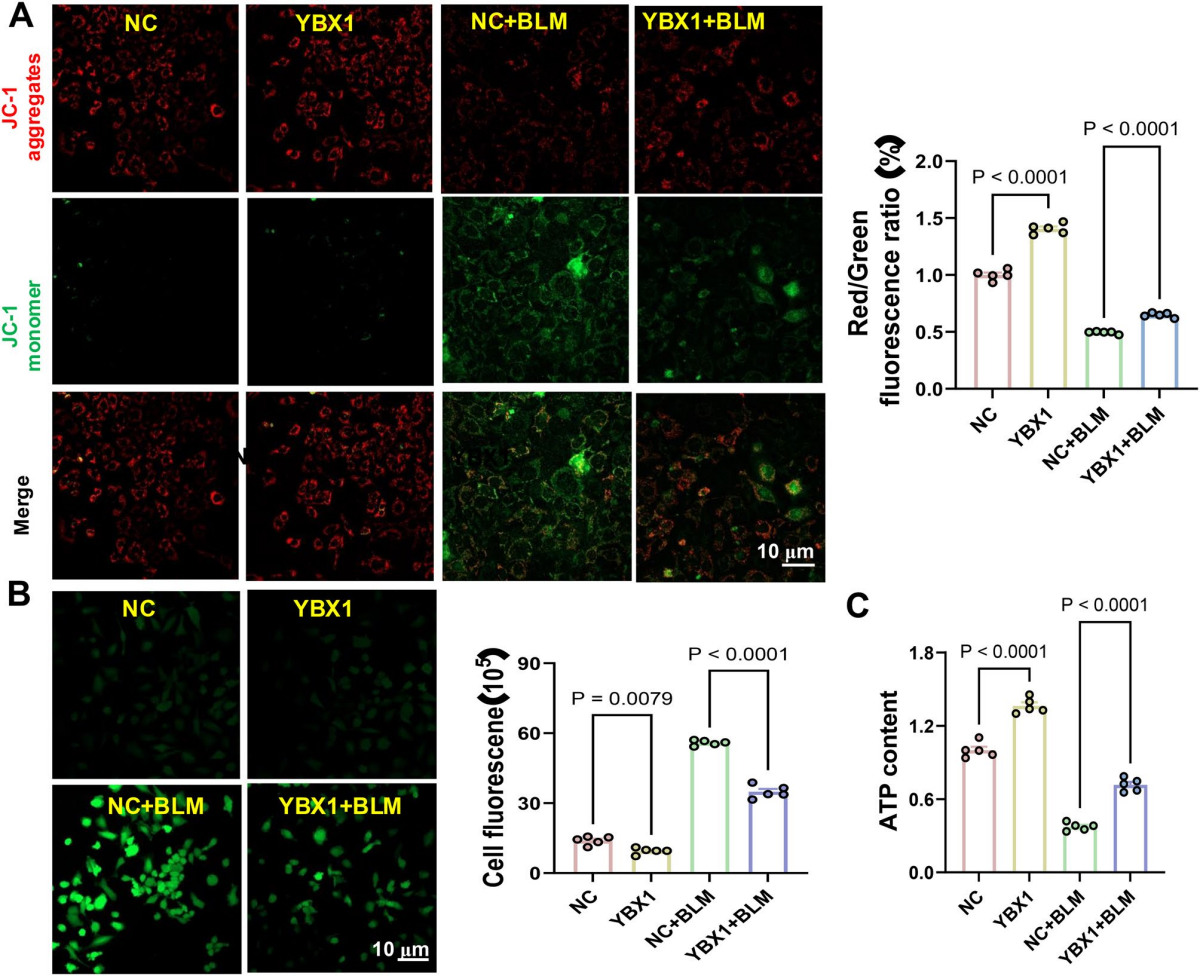
Investigating the role of YBX1 Overexpression in cellular response to oxidative stress. **A**: mitochondrial membrane potential analysis using JC-1 dye. This figure shows mitochondrial membrane potential in cells overexpressing YBX1 and control cells under both normal (NC) and bleomycin (BLM) treatment conditions. Red fluorescence (JC-1 aggregates) indicates healthy, intact mitochondrial membranes, while green fluorescence (JC-1 monomers) signals depolarization. The higher red to green fluorescence ratio in cells overexpressing YBX1 suggests better mitochondrial integrity under oxidative stress. **B**: reactive oxygen species (ROS) generation. This image captures the fluorescence intensity indicating ROS production in cells. Overexpression of YBX1 in cells treated with bleomycin shows maintained fluorescence intensity, suggesting that YBX1 overexpression may mitigate ROS generation and contribute to oxidative stress resilience. **C**: ATP content measurement. The graph displays ATP levels in cells post-treatment, illustrating that YBX1 overexpression provides some protection against bleomycin-induced ATP depletion, allowing cells to maintain higher energy levels under stress

**Fig. 12:**
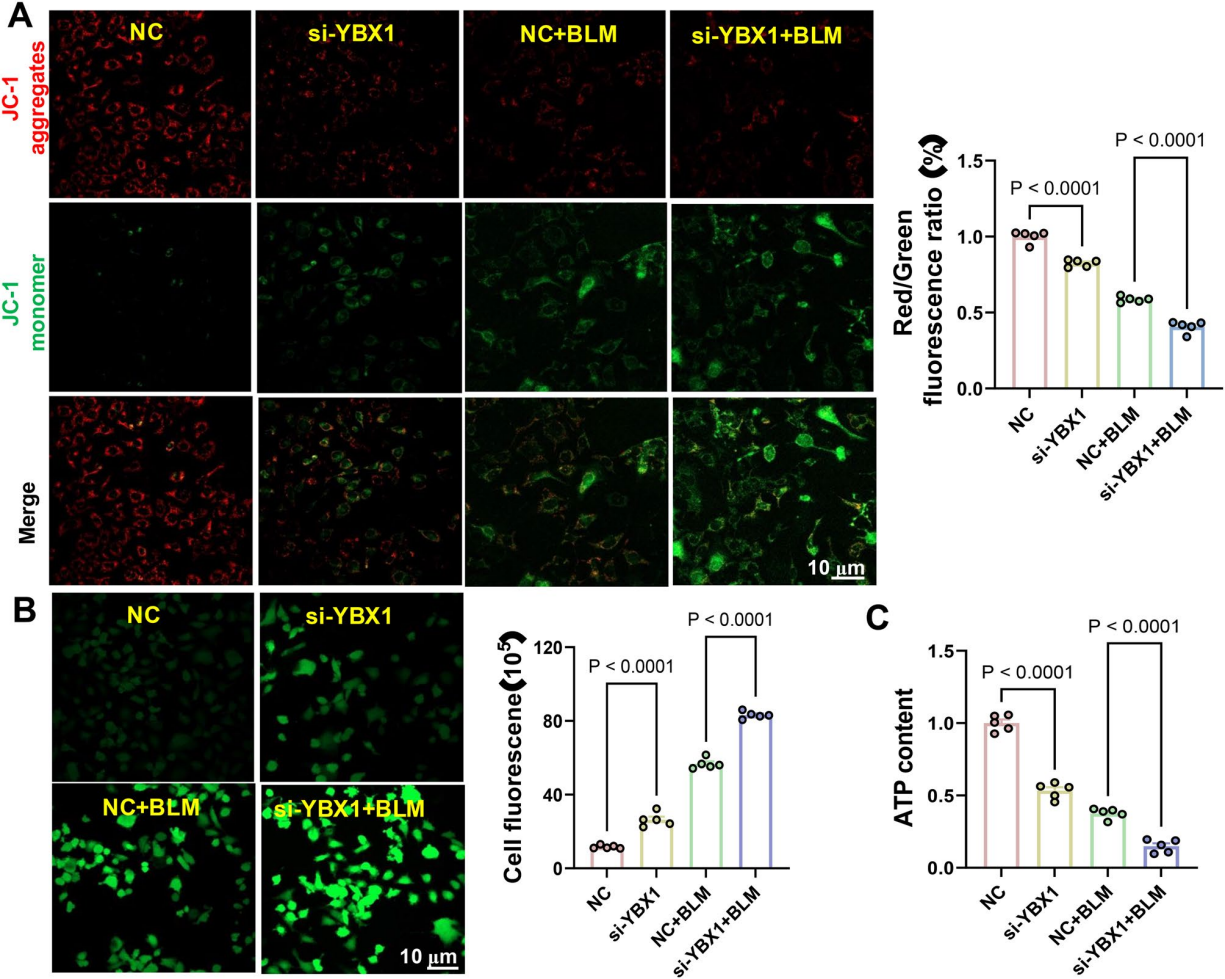
Exploring the consequences of YBX1 knockdown on cellular oxidative stress responses **A**: mitochondrial membrane potential analysis using JC-1 dye. This figure illustrates mitochondrial membrane potential in cells with YBX1 knockdown (si-YBX1) under normal and bleomycin treatment conditions. Increased green fluorescence in si-YBX1 + BLM treated cells highlights enhanced mitochondrial depolarization, indicating significant mitochondrial dysfunction. **B**: reactive oxygen species (ROS) generation. This panel depicts fluorescence imaging of ROS production in cells. With YBX1 knockdown, particularly after bleomycin treatment, there is a marked increase in fluorescence intensity, reflecting heightened ROS production. This increase signals increased oxidative stress and cellular damage, underscoring the protective role of YBX1 against oxidative stress. **C**: ATP content measurement. This graph quantifies ATP levels, showing a significant drop in cells with YBX1 knockdown after bleomycin treatment. The decline emphasizes YBX1‘s crucial role in supporting ATP synthesis and overall mitochondrial resilience, especially under oxidative stress conditions

Effect of YBX1 on Mitochondrial Health in AT2 Cells: Using JC-1 staining, we evaluated mitochondrial membrane potential (MMP) to assess mitochondrial function. In AT2 cells overexpressing YBX1, red fluorescence remained predominant following bleomycin treatment (Fig. 11A), indicating preserved MMP and mitochondrial integrity. In contrast, YBX1 knockdown resulted in a shift to green fluorescence, reflecting significant MMP loss and mitochondrial dysfunction (Fig. 12A). These findings confirm the protective effect of YBX1 on mitochondrial stability under oxidative stress.

Effect of YBX1 on ROS Generation in AT2 Cells: ROS levels were measured to assess oxidative stress. YBX1-overexpressing cells exhibited significantly reduced ROS accumulation after bleomycin exposure (Fig. 11B), indicating that YBX1 attenuates oxidative stress by limiting free radical production. Conversely, ROS levels markedly increased in YBX1-deficient cells under the same conditions (Fig. 12B), underscoring YBX1’s regulatory role in redox balance and oxidative damage mitigation.

Effect of YBX1 on ATP Production in AT2 Cells: ATP quantification provided a readout of cellular energy metabolism. YBX1-overexpressing AT2 cells retained higher ATP levels following bleomycin treatment compared to controls (Fig. 11C), suggesting a protective role for YBX1 in preserving energy homeostasis. In contrast, ATP levels significantly declined in YBX1-deficient cells under the same conditions (Fig. 12C), further demonstrating the importance of YBX1 in maintaining mitochondrial function and energy reserves.

Collectively, these results demonstrate that YBX1 exerts a pivotal protective function in AT2 cells under oxidative stress conditions. By maintaining mitochondrial membrane potential, suppressing ROS generation, and sustaining ATP production, YBX1 enhances the oxidative stress tolerance of AT2 cells.

### Computer-aided virtual drug screening

This study employs computer-aided virtual drug screening technology to systematically evaluate the FDA-approved drug library from the DrugBank database, aiming to identify small molecular compounds that can target and activate the YBX1 protein. The initial screening library included 2,648 small-molecule drugs from the DrugBank database. After data preprocessing (including format conversion and integrity checks), 2,496 valid drug molecules were retained for subsequent analysis. This method offers significant advantages over traditional approaches: firstly, computer simulations enable rapid screening of thousands of known drugs, significantly shortening the research and development cycle; secondly, these drugs have already passed FDA safety reviews, allowing them to progress directly to clinical validation; most importantly, this technology allows for precise atomic-level analysis of the interaction between drugs and YBX1, providing crucial insights for the development of novel anti-pulmonary fibrosis drugs. Docking results (Supplementary Data Table 7) revealed that Omaveloxolone (binding energy: −16.24 kcal/mol) exhibited the strongest YBX1-binding ability among all tested molecules. The binding mode analysis indicated that this molecule stably occupies the active region of YBX1, possibly activating the protein function through an allosteric effect.

### Molecular dynamics simulations

To further validate the binding capacity of Omaveloxolone to YBX1 identified through virtual screening, a 100 ns molecular dynamics simulation of the Omaveloxolone-YBX1 complex was performed. Multidimensional parameter analyses of the simulation trajectory, including RMSD, Rg, centroid distance, hydrogen bonds, buried SASA, free energy landscape, and MM-PBSA binding energy, were conducted to systematically assess the stability and binding mechanism of the complex, providing theoretical support for its potential as a YBX1 activator.

#### RMSD analysis

**Fig. 13:**
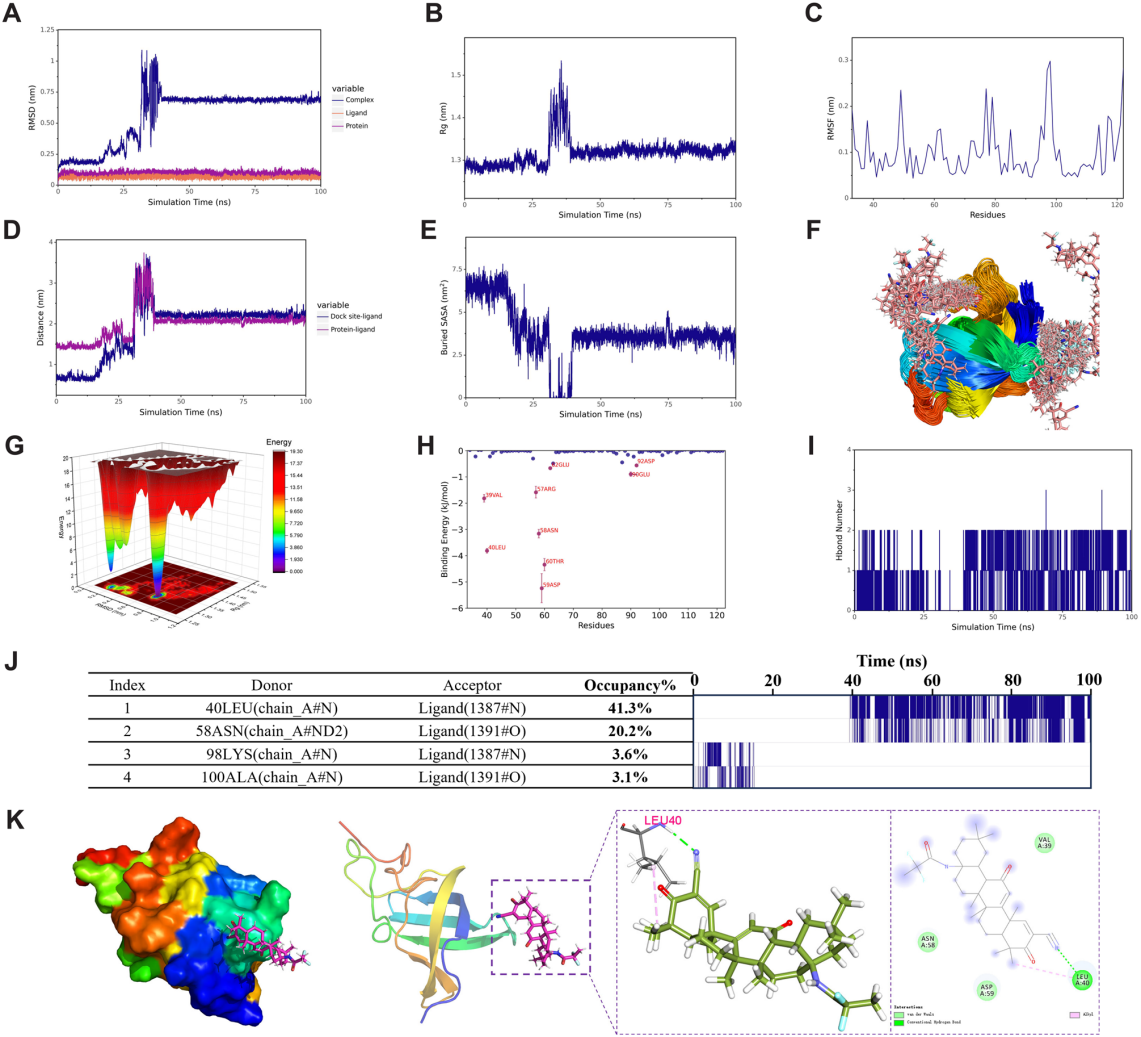
Molecular dynamics simulation results of the YBX1–omaveloxolone complex **A:** RMSD of the complex over 100 ns, indicating structural deviation and equilibration phase; **B:** Radius of gyration (Rg) of the complex, reflecting overall compactness and stability; **C:** RMSF profile of protein residues, identifying flexible and rigid regions during the simulation; **D:** Distance between the ligand centroid and the protein centroid or binding site centroid, showing ligand movement and rebinding behavior; **E:** Buried solvent-accessible surface area (Buried SASA), representing the buried interface area between protein and ligand; **F:** Superposition of representative ligand conformations on the protein surface, illustrating two major binding positions before and after 40 ns; **G:** Free energy landscape (FEL) of the complex constructed using RMSD and Rg as reaction coordinates, indicating two local minima and one global minimum energy state; **H:** MM-PBSA energy decomposition analysis, showing per-residue contributions to the total binding free energy and identifying key interacting residues; **I:** Number of hydrogen bonds between ligand and protein over time, indicating bonding dynamics and stability; **J:** Hydrogen bond frequency analysis, with left panel showing donor–acceptor pairs and occupancy, and right panel visualizing formation frequency as density plots; **K:** Key interactions between ligand and protein residues in a stable conformation, including hydrogen bonds, alkyl interactions, and van der Waals forces

Root Mean Square Deviation (RMSD) is a common measure of structural differences between two molecules, calculated by comparing the spatial coordinates of corresponding atoms. It reflects the conformational similarity between the two molecules and tracks the structural changes of the complex during the simulation, observing whether the changes stabilize. A lower RMSD indicates greater structural similarity. As shown in Fig. 13A, the RMSD of the complex fluctuated significantly between 0 and 40 ns, suggesting that the small molecule gradually dissociated from the initial binding site and re-engaged with the protein. After 40 ns, the RMSD stabilized, indicating that the small molecule binding to the protein reached equilibrium.

#### Rg analysis

The radius of gyration (Rg) is the root mean square distance of all atoms in the molecule relative to its center of mass and reflects the distribution of atoms within the molecule. It is an important parameter for measuring the compactness of protein-small molecule complexes. Smaller Rg values indicate a more compact structure, while larger values suggest a looser structure. As shown in Fig. 13B, the Rg of the complex stabilized after 40 ns, indicating that the structure of the complex remained stable.

#### RMSF analysis

Root Mean Square Fluctuation (RMSF) quantifies the flexibility of individual amino acid residues in a protein by analyzing the extent of their fluctuations along the simulation trajectory. It is a key parameter for identifying flexible and rigid regions within the molecule. As shown in Fig. 13C, RMSF was used to evaluate the local dynamic properties of the protein.

#### Centroid evolution analysis

To analyze the dynamic behavior of the small molecule on the protein surface, the distance changes between the small molecule’s centroid and the centroids of both the initial binding site residues and the protein’s overall center of mass were examined. As shown in Fig. 13D, significant fluctuations in the distance between the small molecule and the protein’s center, as well as between the molecule and the binding site, occurred before 40 ns, indicating that the small molecule detached from the initial binding site. After 40 ns, these distances gradually stabilized, suggesting that the small molecule’s binding to the protein had become stable.

#### Buried SASA analysis

The buried solvent-accessible surface area (Buried SASA) evaluates the extent to which molecular regions are buried within the complex and are inaccessible to solvent. Larger Buried SASA values indicate stronger interactions and larger contact areas between molecules. As shown in Fig. 13E, Buried SASA stabilized after 40 ns, indicating that the contact area between the small molecule and the protein became stable, leading to a more stable binding.

#### Binding conformational overlay and free energy landscape

The simulation trajectory was processed and the conformations were overlaid, as shown in Fig. 13F. The small molecule was dispersed across the protein surface, with two primary binding sites identified, corresponding to the conformational states before and after 40 ns. The free energy landscape (FEL) was constructed using the RMSD and Rg of the complex as reaction coordinates, as shown in Fig. 13G. Two local energy minima were observed, corresponding to the small molecule’s dissociation from the initial binding site and re-binding. The final system reached a global energy minimum, indicating that the conformation stabilized after 40 ns.

#### Hydrogen bond interaction analysis

Hydrogen bonds are one of the key forces in protein-ligand binding and are closely related to electrostatic interactions, reflecting binding strength. As shown in Fig. 13I, the number of hydrogen bonds between the small molecule and the protein fluctuated between 0 and 2. Hydrogen bond frequency analysis revealed that the small molecule formed high-frequency hydrogen bonds with specific amino acid residues at the binding site, likely playing a critical role in the binding process. Fig. 13J(left panel) shows the donors, acceptors, and occupancy of hydrogen bond pairs, while the right panel displays the hydrogen bond formation frequency, with denser lines indicating higher frequencies. The results suggest that hydrogen bond interactions stabilized after 40 ns.

#### Binding energy analysis

For the simulation trajectories during the stable phase, MM-PBSA (Molecular Mechanics-Poisson Boltzmann Surface Area) calculations were performed to determine the binding energy, taking into account factors such as RMSD, Rg, distance, Buried SASA, and interaction energies. The calculation results (Table 1) showed that the ΔEMMPBSA between the small molecule and the protein was −44.862 ± 2.085 kJ/mol, indicating strong binding affinity. In the component energy analysis, the van der Waals interaction energy (ΔEvdw) was higher than the electrostatic interaction energy (ΔEele), with electrostatic and hydrophobic interactions (ΔEnonpol) being comparable. This suggests that van der Waals forces contribute most significantly to the binding, with electrostatic and hydrophobic interactions playing secondary roles. The binding energy decomposition analysis is presented in Fig. 13H, where key amino acids contributing significantly to the binding energy include ASP-59 and THR-60.

**Table 1:** Binding energy and its components under steady-state conditions (kJ/mol)

Complex	ΔE_vdw_	ΔE_ele_	ΔE_pol_	ΔE_nonpol_	ΔE_MMPBSA_	-TΔS	ΔG_bind_*
Protein-Ligand	−74.257±2.432	−14.76±1.429	53.913±1.82	−9.758±0.189	−44.862±2.085	18.105±3.313	−26.757±5.397

*ΔG_bind_=ΔE_vdw_+ΔE_ele_+ΔE_pol_+ΔE_nonpol_-TΔS

#### Structural analysis

Conformations from the stable phase of the simulation were selected for structural analysis. As shown in Fig. 13K, the small molecule forms hydrogen bonds with LEU-40, which also contributes to alkyl hydrophobic interactions. Other residues such as VAL-39, ASN-58, and ASP-59 participate in van der Waals interactions. These non-covalent interactions collectively stabilize the ligand’s binding to the binding site.

## Discussion

Idiopathic pulmonary fibrosis (IPF) is a chronic and progressive interstitial lung disease of unknown etiology, characterized by devastating pulmonary fibrosis and gradual loss of lung function, posing a severe threat to patients’ lives [65]. Currently, treatment options for IPF are extremely limited, and existing drugs have failed to effectively halt disease progression, resulting in poor patient prognosis. Consequently, elucidating the molecular mechanisms underlying IPF and its immunological features has become a critical scientific challenge that requires urgent attention. This study integrates multi-omics data and machine learning algorithms to systematically identify key feature genes and their potential molecular regulatory mechanisms in IPF, with a particular focus on YBX1, a critical regulatory factor. The goal is to provide theoretical foundations for the precision diagnosis, treatment, and drug development for IPF.

### YBX1 as a key regulatory factor in IPF

In the present study, through integrative analysis of multiple transcriptomic datasets and a comprehensive machine learning framework, we identified a set of seven feature genes, among which YBX1 emerged as the most prominent regulator. The glmBoost + Lasso model yielded consistently high diagnostic accuracy across training and validation cohorts, with a mean AUC of 0.945, underscoring the robustness of our findings.

Beyond its diagnostic performance, our functional enrichment analysis revealed that YBX1 was significantly enriched in biological processes central to IPF pathogenesis, including RNA splicing, oxidative stress response, apoptotic signaling, and cytokine-mediated immune regulation. Prior studies provide mechanistic support for this association. For example, YBX1 has been reported to regulate mRNA stability, translational control, and stress granule formation, thereby influencing apoptosis and stress responses [66]. Recent work further identified a role of extracellular YBX1, in cooperation with progranulin, in interfering with TNF binding to its receptor TNFR1, highlighting its potential involvement in inflammatory and apoptotic signaling [60]. In addition, YBX1 and **TIA1** have been implicated in **Fas receptor alternative splicing**, linking these regulators to apoptosis via Fas-mediated pathways [62, 63]. From an immunological perspective, **CFH** plays a recognized role in complement regulation, with downstream effects on cytokine signaling and monocyte recruitment [64]. In particular, the upregulation of apoptotic mediators (e.g., CASP3, FAS, TNFRSF10B), chemotactic factors (e.g., CCL2, CXCL8), and extracellular matrix remodeling genes (e.g., COL1A1, HAS2, MMP2) points to a multi-faceted regulatory axis in which YBX1 may act upstream.

Importantly, these observations are corroborated by Mendelian randomization evidence suggesting a causal protective effect of YBX1 expression on lung function indices such as forced vital capacity (FVC) and forced expiratory volume in one second (FEV₁). This adds a layer of causal inference to our correlative analyses and supports the concept that loss of YBX1 contributes to epithelial dysfunction and maladaptive repair. The clinical relevance of this regulatory role is further highlighted by the RiskScore system derived from the seven feature genes, which effectively stratified patients by age and disease severity. Such stratification provides a promising framework for precision medicine in IPF.

Compared with previous studies [67] that primarily cataloged transcriptional alterations without mechanistic anchoring, our work highlights YBX1 as a central integrator of epithelial survival, immune–inflammatory signaling, and extracellular matrix remodeling. By situating YBX1 at the nexus of these interconnected processes, our study offers novel insight into the molecular architecture of IPF and identifies YBX1 as a compelling candidate for therapeutic targeting.

### YBX1 in AT2 cells and its biological function

AT2 cells are indispensable for maintaining alveolar integrity through surfactant production, epithelial regeneration, and immune regulation. Their dysfunction has long been implicated as a hallmark of idiopathic pulmonary fibrosis (IPF) pathogenesis. In our study, single-cell RNA sequencing revealed a marked reduction in the proportion of AT2 cells in IPF lungs, accompanied by expansion of fibroblasts and macrophages, consistent with the fibrotic remodeling observed in clinical pathology.

Beyond the quantitative loss of AT2 cells, we observed a shift in the distribution of AT2 subtypes, with enrichment of stress-responsive subpopulations (AT2③ and AT2④). Functional enrichment analysis indicated that these subtypes were highly involved in oxidative stress responses, antigen presentation, and epithelial cell differentiation, suggesting that surviving AT2 cells may undergo functional reprogramming to adapt to persistent injurious stimuli [68, 69]. Such reprogramming, while initially compensatory, may paradoxically exacerbate epithelial dysfunction and fibrotic remodeling, reflecting the inability of AT2 cells to sustain effective repair under persistent stress.

Furthermore, YBX1 expression was closely correlated with phenotypic transitions among AT2 subtypes. Within these stress-responsive subpopulations, YBX1 expression was significantly downregulated. Experimental validation in epithelial cells demonstrated that YBX1 is essential for preserving mitochondrial integrity, reducing reactive oxygen species (ROS), and maintaining ATP levels under bleomycininduced stress. Conversely, YBX1 knockdown markedly increased apoptosis and impaired epithelial viability. These observations are consistent with documented roles of YBX1 in maintaining mitochondrial translation fidelity and preventing stressinduced cell death [66, 70].

Taken together, these results suggest that YBX1 acts as a molecular safeguard in AT2 cells, sustaining their viability and functional stability in the face of injurious stimuli. Loss of YBX1 compromises these protective mechanisms, leading to increased epithelial apoptosis and impaired repair capacity, both of which are central to the progression of pulmonary fibrosis.

### YBX1 and its interaction with the immune microenvironment

The immune microenvironment is increasingly recognized as a pivotal contributor to the progression of IPF. Previous studies have shown that alterations in the balance of innate and adaptive immune cells, particularly macrophages, dendritic cells, and lymphocytes, play a central role in sustaining chronic inflammation and promoting fibrotic remodeling [71, 72]. Our immune infiltration analysis revealed substantial alterations in immune cell composition, including increased proportions of naïve B cells, plasma cells, and CD4^+^ memory T cells, accompanied by a reduction in activated dendritic cells, resting NK cells, and neutrophils.

The reduction of activated dendritic cells in IPF is particularly noteworthy, as these cells are a major source of interferon-β (IFN-β), a cytokine with known antifibrotic and antiviral effects. Their depletion likely compromises IFN-β–mediated defense mechanisms, rendering the lung more vulnerable to persistent epithelial injury and aberrant repair [73]. While alveolar epithelial cells, macrophages, and activated T cells have been reported in prior studies as potential alternative IFNβ producers, our dataset does not allow direct quantification of their contribution. Thus, although compensatory mechanisms may exist, the overall signaling strength appears insufficient to mount an effective antifibrotic response [74]. This imbalance provides a plausible mechanistic explanation for the chronic inflammatory milieu characteristic of IPF.

In addition, we observed a reduction in neutrophils in IPF lungs. Although increased neutrophil activity is frequently associated with acute lung injury and early inflammatory responses, the lower neutrophil proportion in our analysis may reflect the transition to a chronic fibrotic stage, where macrophages and lymphocytes become the predominant drivers of the immune response. It is also possible that age-related differences, as our IPF cohort was significantly older than the controls, contribute to this observation. Together, these results suggest a shift from acute neutrophil-dominated inflammation toward a microenvironment in which macrophages and their associated signaling pathways play a central role.

Consistent with this shift, our intercellular communication analysis revealed a striking upregulation of complement signaling, particularly through the C3 axis, in IPF lungs. Of note, YBX1 expression was significantly reduced in both AT2 cells and tissue-resident alveolar macrophages (TR-AMs), coinciding with marked upregulation of C3. This reciprocal regulation suggests that loss of YBX1 may contribute to the upregulation of C3 observed in TR-AMs and AT2 cells, thereby amplifying complement-driven immune activation. Complement C3, in turn, facilitates crosstalk between AT2 cells and M2 macrophages, promoting fibroblast activation and extracellular matrix deposition. These findings are consistent with recent reports implicating complement activation as a driver of fibrogenesis [75, 76].

Importantly, supplementary analysis of a bleomycin-induced pulmonary fibrosis mouse model revealed dynamic remodeling of alveolar macrophages, with expansion of Mo-Ams at early stages and partial recovery of TR-AMs at later stages. Within TR-AMs, Ybx1 downregulation was accompanied by C3 upregulation, mirroring the pattern observed in human IPF samples. This concordance underscores the biological relevance of the YBX1–C3 axis as a mechanistic link between epithelial injury, macrophage reprogramming, and sustained complement activation.

Collectively, these findings suggest that YBX1 functions as a pivotal regulator of epithelial–immune communication in IPF. Its downregulation may initiate a cascade of events, including impaired IFN-β signaling, enhanced complement activation, and recruitment of pro-fibrotic immune populations, thereby perpetuating a cycle of chronic inflammation and progressive fibrosis. Nevertheless, we acknowledge that our analysis did not directly measure the active complement fragment C3a, nor did it quantify the precise functional contribution of each cellular source of complement. These limitations mean that while our data strongly support a YBX1–C3 axis, the downstream effector mechanisms should be interpreted with caution and warrant further experimental validation. Targeting the YBX1–C3 axis may thus represent a promising therapeutic strategy to restore immune homeostasis and mitigate fibrotic progression.

### Significance of virtual drug screening and molecular dynamics simulations

Therapeutic targeting of YBX1 remains a largely unexplored area in idiopathic pulmonary fibrosis (IPF). Current antifibrotic drugs, such as pirfenidone and nintedanib, primarily delay disease progression rather than reversing established fibrosis, underscoring the urgent need for novel therapeutic strategies. In this context, structure-based drug design offers a promising avenue for identifying small-molecule modulators of key regulatory factors like YBX1 [66].

Leveraging the structural model of YBX1, we employed virtual drug screening coupled with molecular dynamics (MD) simulations to identify candidate compounds with high binding affinity and favorable interaction stability. Among the screened molecules, Omaveloxolone demonstrated particularly strong and stable binding within the nucleic acid–interacting domain of YBX1. Conformational analysis revealed that Omaveloxolone may enhance YBX1’s transcriptional regulatory capacity by stabilizing its nucleic acid-binding configuration.

From a mechanistic perspective, activation of YBX1 by Omaveloxolone could help preserve AT2 cell homeostasis by improving mitochondrial integrity, reducing oxidative stress, and preventing apoptosis—processes previously linked to epithelial injury in IPF. This pharmacological effect may extend beyond epithelial cells, indirectly attenuating aberrant immune–epithelial crosstalk and limiting pro-fibrotic macrophage activation, as suggested by our findings on the YBX1–C3 axis.

It is noteworthy that Omaveloxolone has been investigated in clinical settings for its antioxidant and anti-inflammatory properties, including in mitochondrial and neurodegenerative disorders. Such as Friedreich’s ataxia, where it demonstrated significant improvements in mitochondrial function and cellular redox balance [77, 78]. Its pharmacological profile—particularly its ability to modulate Nrf2 signaling and oxidative stress pathways [79–81]—aligns well with the pathological mechanisms observed in IPF, thereby supporting its rationale as a candidate for repurposing. Nevertheless, rigorous in vitro and in vivo validation will be required to confirm its efficacy and clarify the extent to which it directly modulates YBX1 activity in pulmonary fibrosis.

Taken together, our integrative computational approach provides a strong proof of concept that structure-based screening and MD simulations can accelerate the identification of novel YBX1-targeting agents. Omaveloxolone emerges as a promising lead compound, offering both mechanistic plausibility and translational potential. These findings not only broaden the therapeutic landscape for IPF but also establish a framework for rational drug discovery targeting key molecular regulators of fibrotic progression.

## Conclusion

This study systematically investigates the potential role of YBX1 in the pathogenesis of idiopathic pulmonary fibrosis (IPF) through multi-omics data integration, machine learning modeling, and molecular simulations. First, the risk prognostic model constructed based on YBX1 feature genes demonstrates excellent predictive performance and clinical utility, highlighting the importance of YBX1 in disease progression assessment. Further analysis reveals dynamic expression patterns of YBX1 in different AT2 cell subpopulations and their developmental trajectories, suggesting that YBX1 may play a key regulatory role in cell fate determination. Additionally, YBX1 regulates mitochondrial function and ROS production in AT2 cells, influencing their survival status. Downregulation of YBX1 may disrupt cell proliferation, differentiation, and interactions with immune cells, contributing to the onset and progression of IPF.

In the search for potential therapeutic targets, we identified several small molecules with high binding affinity to YBX1 using virtual drug screening and molecular dynamics simulations. Omaveloxolone is predicted as a potential YBX1 activator, demonstrating good binding stability and conformational regulation. Although direct experimental evidence for its activation effect is currently lacking, this study provides a structural basis and theoretical support for future experimental validation and clinical translation.

Overall, this research not only deepens our understanding of the role of YBX1 in IPF pathogenesis but also lays a solid foundation for its development as a therapeutic target. By integrating bioinformatics, single-cell omics, and structural biology strategies, we have established a systematic framework for exploring new targets and drug screening, providing novel insights and theoretical support for the precision treatment of IPF, a complex lung disease.

## Electronic supplementary material

Below is the link to the electronic supplementary material.

**Supplementary Material 1:** Supplementary Figure 1

**Supplementary Material 2:** Supplementary Table 1

**Supplementary Material 3:** Supplementary Table 2

**Supplementary Material 4:** Supplementary Table 3

**Supplementary Material 5:** Supplementary Table 4

**Supplementary Material 6:** Supplementary Table 5

**Supplementary Material 7:** Supplementary Table 6

**Supplementary Material 8:** Supplementary Table 7

## Data Availability

The datasets used and analyzed in this study are referenced within the paper. The R, Python, and Perl scripts employed in the analysis are available from the corresponding authors upon reasonable request.
