# Supplementary material for: YBX1 regulation of alveolar type II epithelial cells in idiopathic pulmonary fibrosis: mechanistic insights and small-molecule drug screening
Source: J Transl Med. 2025 Nov 18;23:1301. doi: 10.1186/s12967-025-07297-2 (PMC12625311; doi:10.1186/s12967-025-07297-2)
Supplement: Supplementary file 1 — Supplementary Material 1: Supplementary Figure 1 [file 12967_2025_7297_MOESM1_ESM.docx]

**
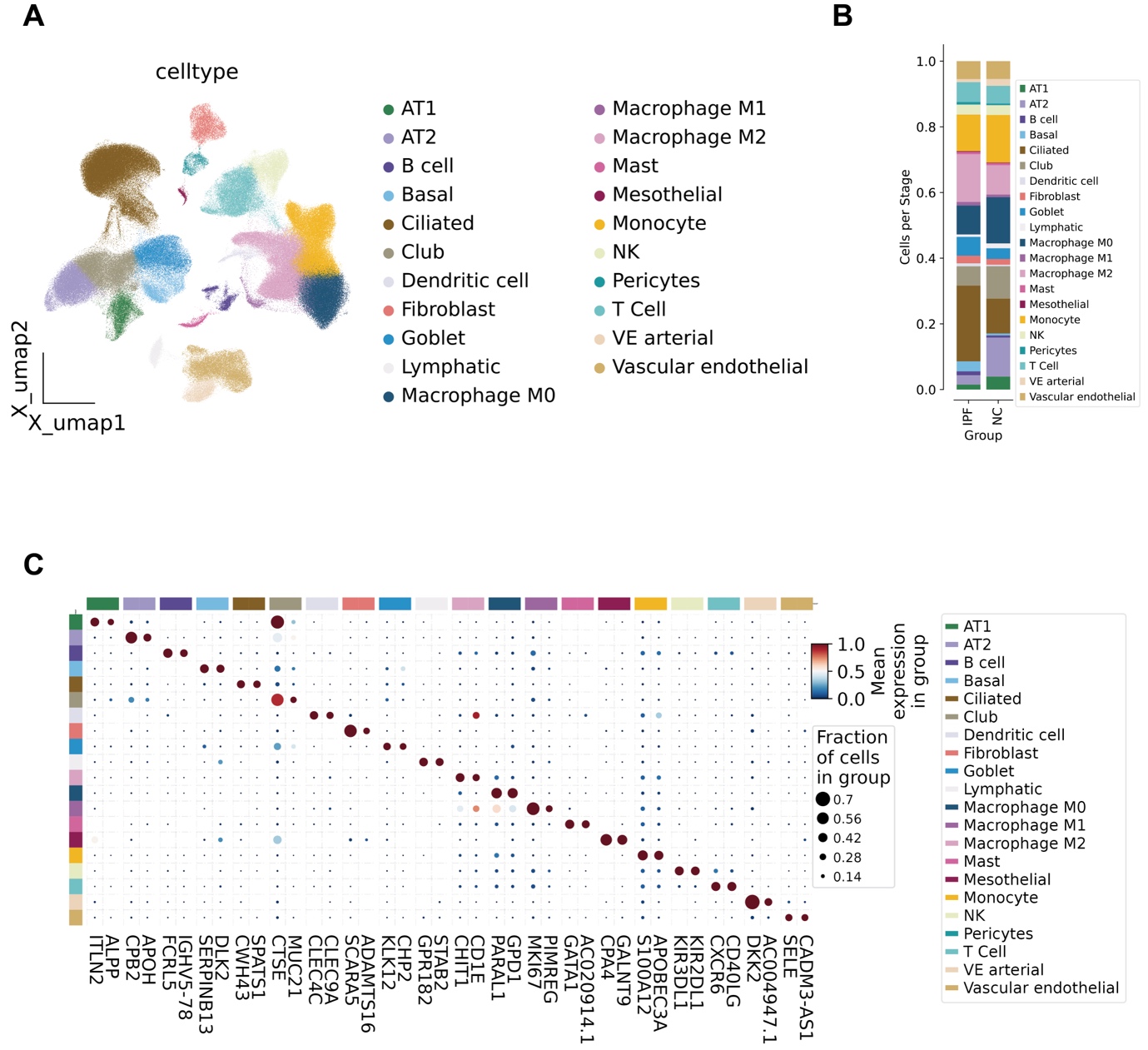
**

**FigureS1 A:** UMAP plot displaying the distribution of different cell types in the dataset, color-coded as indicated in the legend. Each cluster represents a distinct cell type based on gene expression profiles. **B:** Stacked bar plots illustrating the proportions of various cell types in the IPF and NC groups. Noticeable shifts in cellular composition are observed in the IPF group, particularly in macrophages and fibroblasts. **C:** Dot plot depicting the expression of key genes across different cell types. The size of the dots indicates the fraction of cells expressing each gene, and the color represents the mean expression level within each cell type.
